# Metabolic adaptation to acute metabolic stress via PFKFB3 upregulation in rodent beta cells

**DOI:** 10.3389/fendo.2025.1552700

**Published:** 2025-06-17

**Authors:** Koki Chiba, Hiroshi Nomoto, Rimi Izumihara, Xinxin Zhang, Hiraku Kameda, Akinobu Nakamura, Tatsuya Atsumi

**Affiliations:** ^1^ Department of Rheumatology, Endocrinology and Nephrology, Faculty of Medicine and Graduate School of Medicine, Hokkaido University, Sapporo, Japan; ^2^ Division of Endocrinology, Metabolism, and Rheumatology, Department of Internal Medicine, Asahikawa Medical University, Asahikawa, Japan

**Keywords:** beta cells, diabetes, metabolic remodeling, glycolysis, PFKFB3

## Abstract

**Introduction:**

Pancreatic beta cells undergo metabolic remodeling in response to metabolic overload, but the functional significance of this remains unclear. 6-phosphofructo-2-kinase/fructose-2,6-biphosphatase 3 (PFKFB3) is a glycolytic regulator that may play a role in beta cell adaptation under acute metabolic stress. This study aimed to investigate the involvement of PFKFB3 in beta cell function under such stress.

**Methods:**

INS-1 832/13 cells and mouse-derived pancreatic islets were cultured under varying glucose concentrations. Male ob/+ and ob/ob mice were assigned to ad libitum feeding, restricted feeding, or sodium–glucose cotransporter 2 inhibitor (SGLT2i) treatment groups. Glucose tolerance, insulin secretion, and expression of metabolism-related genes were assessed. Knockdown of PFKFB3 and pharmacological inhibition of glycolysis were used to evaluate its functional role; MTT assays were conducted to assess cellular metabolic activity.

**Results:**

Exposure to high glucose concentrations and excessive metabolic demand resulted in the upregulation of PFKFB3 expression *in vitro* and *in vivo*. Interventions such as restricted feeding and SGLT2i administration partially reduced metabolic stress-associated PFKFB3 upregulation in ob/ob mice. Knockdown of PFKFB3 or pharmacological inhibition of glycolysis resulted in decreased insulin secretion and impaired glucose tolerance. MTT assay results showed a time-dependent reduction in metabolic activity following *Pfkfb3* knockdown, suggesting compromised cell survival under acute metabolic stress.

**Conclusion:**

PFKFB3 upregulation under acute metabolic stress may be an adaptive response that helps maintain beta cell function. Suppression of PFKFB3 activity compromises insulin secretion and glucose tolerance, highlighting the importance of this pathway in metabolic adaptation to transient stress.

## Introduction

The reduction in insulin secretion in diabetes results from decreases in both the function and number of pancreatic beta cells. Investigating the mechanisms that regulate these changes is therefore important for understanding diabetes pathophysiology (1–4). Previous reports suggest that glucose is metabolized into pyruvate and subsequently utilized in the tricarboxylic acid (TCA) cycle and oxidative phosphorylation in the pancreatic islets of non-diabetic rats. This process leads to substantial production of adenosine triphosphate (ATP), which contributes to insulin secretion (5, 6). However, in the pancreatic islets of diabetic rats, glycolysis activation occurs via the hypoxia-inducible factor (HIF)1α/6-phosphofructo-2-kinase/fructose-2,6-biphosphatase 3 (PFKFB3) pathway had been reported (7, 8).

PFKFB3 is a master regulator of the glycolytic pathway regulated by HIF1α (9). Under certain conditions that lead to a decline in TCA cycle activity within the cell, stabilized HIF1α leads to an increase in PFKFB3 expression. Subsequently, PFKFB3 synthesizes fructose-2,6-bisphosphate and activates the glycolytic enzyme phosphofructokinase-1, promoting the utilization of intracellular glucose in the glycolytic pathway (10). Under metabolic stress conditions such as glucotoxicity, pancreatic beta cells undergo various metabolic stresses that induce mitochondrial dysfunction, thereby reducing oxidative phosphorylation. Activation of PFKFB3 appears to be involved in these intracellular metabolic changes, which result in the primary ATP-generating pathway of pancreatic beta cells switching from oxidative phosphorylation to glycolysis (11). Indeed, PFKFB3-mediated metabolic remodeling of beta cells has been observed in both experimental animals and samples from humans with type 1 and type 2 diabetes (8, 12). However, whether these intracellular metabolic changes can be corrected through diabetes-improving interventions remains unclear. Additionally, the significance of these intracellular metabolic changes, particularly whether they contribute to the improvement or exacerbation of diabetes, or are merely a result of the diabetic state, is not fully understood.

Therefore, this study aimed to investigate whether intracellular metabolic remodeling in pancreatic beta cells under acute metabolic stress is reversible, and to evaluate its functional significance in the context of early-stage stress adaptation.

## Methods

### Animals

We used 6-week-old, male, BKS.B6.Cg-*Lep^ob^*/J(ob/ob), ob/+, C57BL/6J mice purchased from Oriental Yeast Co. Ltd. (Tokyo, Japan). Each mouse was housed individually for all experiments under controlled ambient conditions with a 12-h light/dark cycle (lights on at 7 am). Animals were given free access to drinking water and the temperature was maintained at 25°C. The mice were euthanized by cervical dislocation after isoflurane exposure. The study is reported in accordance with ARRIVE guidelines. No human subjects were recruited for this study. The study protocol was approved by the Animal Use Committee of Hokkaido University Graduate School of Medicine and was conducted in compliance with the Animal Use Guidelines of Hokkaido University.

### Diet protocol

Standard chow (MF; Oriental Yeast Co. Ltd., Tokyo, Japan) was used, as described previously (13). Tofogliflozin, a SGLT2i, was provided by Kowa Company, Ltd. (Tokyo, Japan).

### Measurement of biochemical parameters

Blood glucose was measured using a Glutestmint portable glucose meter (Sanwa Chemical Co., Nagoya, Japan). Plasma was separated from blood samples and insulin levels were determined with an insulin ELISA kit (Morinaga Institute of Biological Science, Yokohama, Japan) as described previously (14).

### Oral glucose tolerance test

For the OGTT, mice were fasted for 16 h before being orally loaded with glucose (1.0 mg/g body weight). Blood samples were collected from the tail vein at 0, 15, 30, 60 and 120 min after glucose administration to determine blood glucose levels. The total area under the blood glucose concentration curve (AUC) was determined from 0 to 120 min after glucose loading.

### INS-1 832/13 cell culture

INS-1 832/13 cells purchased from Merck Millipore (Darmstadt, Germany) were cultured in a humidified atmosphere of 5% CO_2_/95% air at 37°C in RPMI-1640 medium supplemented with 10% fetal bovine serum, 1% penicillin–streptomycin, 50 μM β-mercaptoethanol, 1 mM sodium-pyruvate, 10 mM HEPES and 1% GlutaMAX (standard culture medium; all Sigma-Aldrich, St. Louis, MO, USA). Unless otherwise stated, the glucose concentration was 11 mM.

### si-RNA induction

For siRNA interference, siRNA against PFKFB3 (ON-TARGET plus Rat Pfkfb3 siRNA SMART pool, L-095107-02-0010, Horizon Discovery, Cambridge, UK) and its control siRNA (ON-TARGET plus Non-targeting Pool, D-001810-10-20, Horizon Discovery) were transfected into INS cells following the manufacturer’s instructions. After culture for 48 h, cells were collected for analysis.

### MTT Assay

To evaluate the effect of *Pfkfb3* knockdown on cellular metabolic activity, INS-1 832/13 cells were transfected with *Pfkfb3*-specific siRNA and cultured for 24, 48, or 72 hours in 11 mM glucose-containing medium. Cell activity was assessed using an MTT assay kit (Cell Proliferation Kit I, Roche, Cat. No. 11465007001, Sigma-Aldrich), following the manufacturer’s instructions. Absorbance was measured at 570 nm using a microplate reader. This assay reflects mitochondrial metabolic activity and may serve as an indirect indicator of cell viability and/or proliferative potential.

### Beta cell morphology and immunohistochemistry

Isolated pancreatic tissues were immersion-fixed in 4% paraformaldehyde at 4°C overnight. Tissues were paraffin-embedded and 5-μm sections were mounted on glass slides using standard procedures. Sections were immersed for 15 min in methanol containing 0.3% (v/v) H_2_O_2_ to deactivate endogenous peroxidase activity. After rinsing with PBS, sections were immunostained with rabbit anti-human insulin antibody (diluted 1:1000) (Santa Cruz Biotechnology, Santa Cruz, CA, USA). Beta cell mass was calculated as follows: beta cell mass (mg) = (beta cell area / pancreatic area) × pancreas weight (mg) (13, 14).

For immunofluorescence, tissue sections were incubated overnight at 4°C with the primary antibodies listed in **Supplementary Table S1**. After rinsing with PBS, tissues were incubated with secondary antibodies for 60 min using previously described procedures (**Supplementary Table S1**) (14, 15). Immunofluorescence images were acquired using a BZ-II analyzer (Keyence, Osaka, Japan) according to the manufacturer’s instructions.

### Islet isolation

Islets were isolated using collagenase from Clostridium histolyticum (Sigma-Aldrich, St. Louis) according to the manufacturer’s instructions. To measure insulin content, isolated islets were incubated in acid ethanol and the insulin concentration in the assay buffer was measured using an insulin ELISA kit (Morinaga Institute of Biological Science).

### Quantitative real-time PCR

Total RNA was isolated from INS-1 832/13 cells and islets using an RNeasy mini kit (Qiagen) and was used as the starting material for cDNA preparation. Quantitative real-time PCR was performed in duplicate using a 7500 Fast Real Time PCR system with SYBR Green PCR Master Mix (Applied Biosystems, Foster City, CA, USA). The primer sequences used are listed in **Supplementary Table S2**.

### Western blotting

Primary antibodies and secondary antibodies were used as shown in **Supplementary Table S3**. Protein bands were visualized with an ImmunoStar LD western blotting detection kit (Wako Pure Chemical Industries, Osaka, Japan). Images were obtained using an LAS-4000 imager (Fujifilm, Tokyo, Japan), and the specific band intensities were normalized to those of beta-actin. To quantify protein expression, densitometric analysis was performed using ImageJ software (National Institutes of Health, Bethesda, USA).

### Seahorse analysis

Oxygen consumption rate (OCR) measurements were obtained over time (min) using an extracellular flux analyzer. Bioenergetic parameters were obtained by adding glucose (5.5, 11, 22 mM) to derive OCR under stimulated conditions; the ATP synthase inhibitor Oligomycin A to derive ATP-linked OCR; carbonyl cyanide p-trifluoro methoxyphenylhydrazone (FCCP) to uncouple the mitochondria to derive maximal OCR; and mitochondrial complex inhibitor Rotenone/Antimycin A to block mitochondrial respiration.

### Statistical analyses

Data are expressed as mean ± standard deviation (SD). Individual comparisons between more than two groups were assessed using one-way analysis of variance (ANOVA) followed by Bonferroni’s *post-hoc* test. Data were analyzed using JMP Pro v17.0.0 (SAS Institute, Cary, NC, USA). *P <*0.05 was considered statistically significant.

Additional method details are provided in the Supplementary information, including **Supplementary Tables S1**-**3**.

## Results

### Influence of glucose stimulation on intracellular metabolic changes

**Figure 1:**
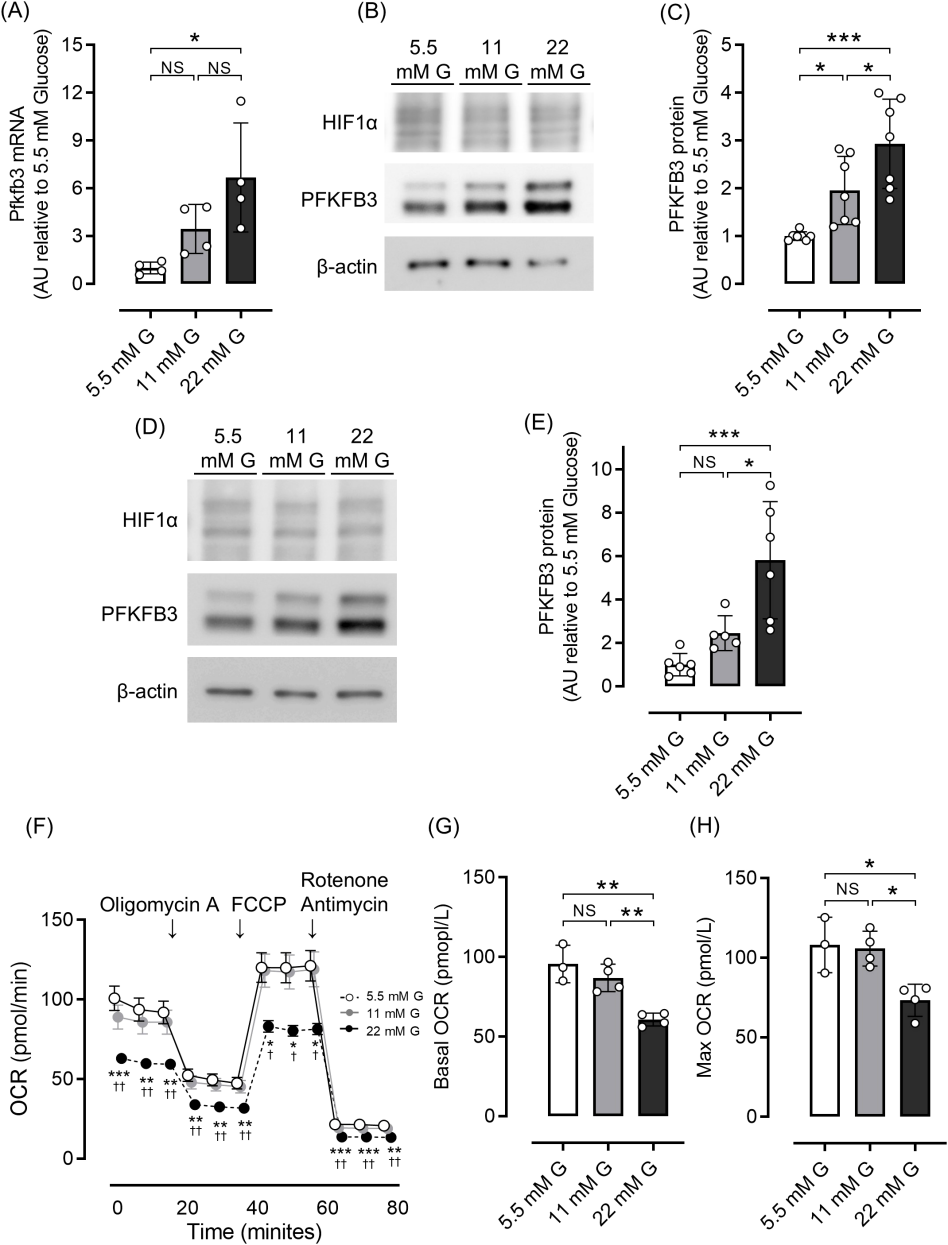
Effects of different glucose concentrations on PFKFB3 expression and mitochondrial function in INS-1 832/13 cells. **(A)** Gene expression of *Pfkfb3* analyzed by quantitative real-time PCR in INS-1 cells collected after being cultured for 48 h in each glucose concentration. Data are normalized to *beta actin* expression. **(B)** Representative Western blot of PFKFB3 and HIF1α protein levels in cell lysate from INS-1 832/13 cells cultured for 48 h in each glucose concentration. The gels and blots were cropped for clarity. For full photos, see **Supplementary Figure S8**. **(C)** Quantification of PFKFB3 expression in INS-1 832/13 cells under different glucose conditions. **(D)** Representative Western blot of PFKFB3 and HIF1α protein levels in pancreatic islets of C57BL6/J mice cultured under different glucose concentrations. The gels and blots were cropped for clarity. For full photos, see **Supplementary Figure S9**. **(E)** Quantification of PFKFB3 expression in islets under different glucose conditions. **(F)** OCR measurements were obtained over time (min) using an extracellular flux analyzer. Bioenergetic parameters were obtained by adding glucose (5.5, 11, 22 mM) to derive OCR under stimulated conditions; the ATP synthase inhibitor Oligomycin A to derive ATP-linked OCR; FCCP to uncouple the mitochondria for maximal OCR; and the mitochondrial complex inhibitor Rotenone/Antimycin A to block mitochondrial respiration. White open circles, 5.5 mM glucose; gray circles, 11 mM glucose; black circles, 22 mM glucose. **(G, H)** Quantification of basal OCR **(G)** and maximal OCR **(H)**. Data are the mean ± SD. N = 4 in **(A)**, 7 in **(C)**, 5–6 in **(E)** and 3–4 in **(F–H)**. * *p <*0.05, ** *p <*0.01, *** *p <*0.001, one-way ANOVA with Bonferroni’s *post hoc* test. AU, arbitrary units; G, glucose; HIF1α, hypoxia inducible factor α; PFKFB3, 6-phosphofructo-2-kinase/fructose-2,6-biphosphatase 3; NS, not significant; OCR, oxygen consumption rate; FCCP, carbonyl cyanide p-trifluoro methoxyphenylhydrazone.

We first examined the effects of different glucose concentrations on intracellular metabolism by evaluating the expression of PFKFB3, a glycolytic master regulator enzyme, using a rodent beta cell line and isolated islets. Quantitative real-time PCR analysis demonstrated significantly higher *Pfkfb3* expression in INS-1 cells in a high-glucose environment than a low-glucose environment (22 mM glucose vs. 5.5 mM glucose, *p <*0.05; **Figure 1A**). Consistent with these results, in both INS-1 832/13 cells and isolated islets, expression of PFKFB3 was significantly higher in the high glucose group (22 mM) than the lower glucose groups (11 and 5.5 mM, all *p <*0.05; **Figures 1B–E**). While expression of PFKFB3 increased in response to high glucose conditions, mitochondrial function assessed by seahorse analysis was significantly lower under high glucose conditions, presumably due to glucose toxicity (22 mM glucose vs. 11 and 5.5 mM glucose, all *p <*0.05; **Figures 1F–H**). These results suggest a close relationship between mitochondrial dysfunction and intracellular shifts in glycolytic energy metabolism.

The ob/ob mouse is an obese diabetic animal model that becomes overweight and hyperglycemic within weeks of birth and progressively worsens with age. Therefore, excessive metabolic load on pancreatic beta cells is assumed in these mice. At 6 weeks of age, when hyperglycemia was not pronounced, PFKFB3 expression in pancreatic beta cells of ob/ob mice was not clearly different from that of control ob/+ mice. However, as the weeks progressed, body weight and hyperglycemia in ob/ob mice increased and higher PFKFB3 expression was observed (**Supplementary Figures S1A, B**).

**Figure 2:**
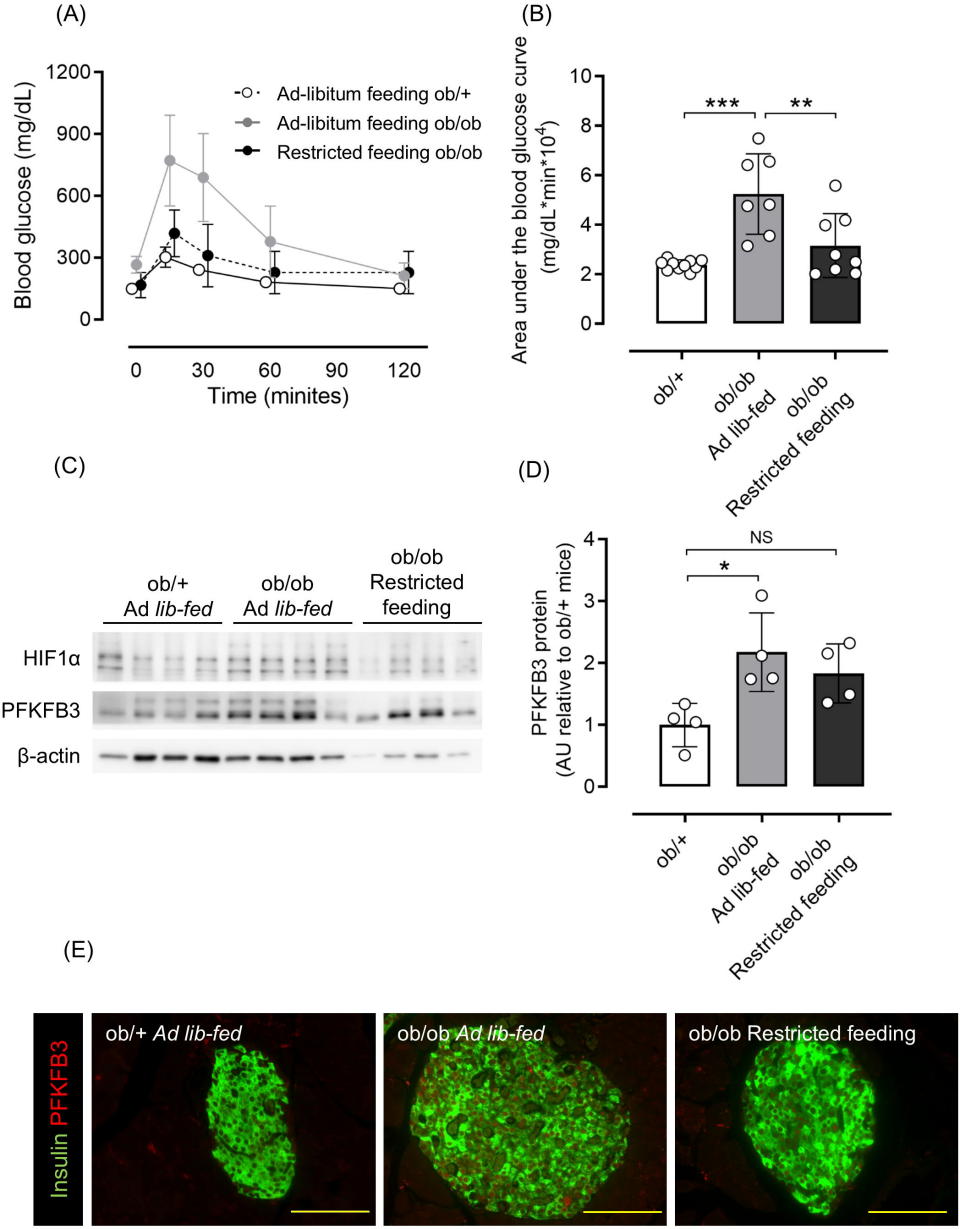
Effects of restricted feeding on glucose tolerance and PFKFB3 expression in pancreatic islets of ob/ob mice. **(A)** Blood glucose changes during the oral glucose tolerance test after being fed a standard chow diet ad libitum or restricted to 2 g/day from 6 to 10 weeks of age. White circles, ad-libitum feeding ob/+ mice; gray circles, ad-libitum feeding ob/ob mice; black circles, restricted feeding ob/ob mice. **(B)** Area under the curve of the oral glucose tolerance test. **(C)** Representative western blot of PFKFB3 and HIF1α protein levels in pancreatic islets of ob/+ and ob/ob mice. The gels and blots were cropped for clarity. For full photos, see **Supplementary Figure S10**. **(D)** Quantification of PFKFB3 expression in islets under different conditions. Data are normalized to β-actin expression. White bar, ad-libitum feeding ob/+ mice; gray bar, ad-libitum feeding ob/ob mice; black bar, restricted feeding ob/ob mice. **(E)** Immunohistochemical staining of insulin and PFKFB3 in pancreatic islets. Red, PFKFB3; green, insulin. Yellow bar; 100 μm. Data are mean ± SD. N = 6–10 in **(A, B)** and 4 in **(D)**. * *p <*0.05, ** *p <*0.01, *** *p <*0.001, one-way ANOVA with Bonferroni’s *post hoc* test. HIF1α, hypoxia inducible factor α; PFKFB3, 6-phosphofructo-2-kinase/fructose-2,6-biphosphatase 3; AU, arbitrary units; NS, not significant.

Considering that excessive food intake contributes to obesity and hyperglycemia, some ob/ob mice had food intake restricted starting at 6 weeks of age. As a result, body weight and blood glucose levels were comparable to those of ob/+ mice (**Supplementary Figures S2A–C**), as was glucose tolerance assessed by an oral glucose tolerance test (OGTT) (**Figures 2A, B**). Western blot analysis of isolated pancreatic islets showed an upregulation of PFKFB3 expression in the ob/ob ad-libitum group compared with the control group (*p <*0.05 for PFKFB3); however, in the islets of mice from the restricted feeding group, the expression of PFKFB3 was suppressed to levels similar to the control group (**Figures 2C, D**). Similarly, in pancreatic sections, the expression of PFKFB3 in beta cells was significantly higher in the ob/ob ad-libitum group than the control group (*p <*0.05); in the restricted feeding group, PFKFB3 expression was significantly lower than in the ob/ob ad-libitum group, but significantly higher than in the control group (both *p <*0.001; **Figure 2E**, **Supplementary Figure S3A**). Insulin staining of pancreatic islets showed a significant compensatory increase in beta cell mass in the ob/ob ad-libitum group (*p <*0.001 vs. control and restricted feeding groups), which was suppressed by food restriction (**Supplementary Figure S3B**).

### Flexibility of intracellular metabolic changes in obese-diabetic pathology

**Figure 3:**
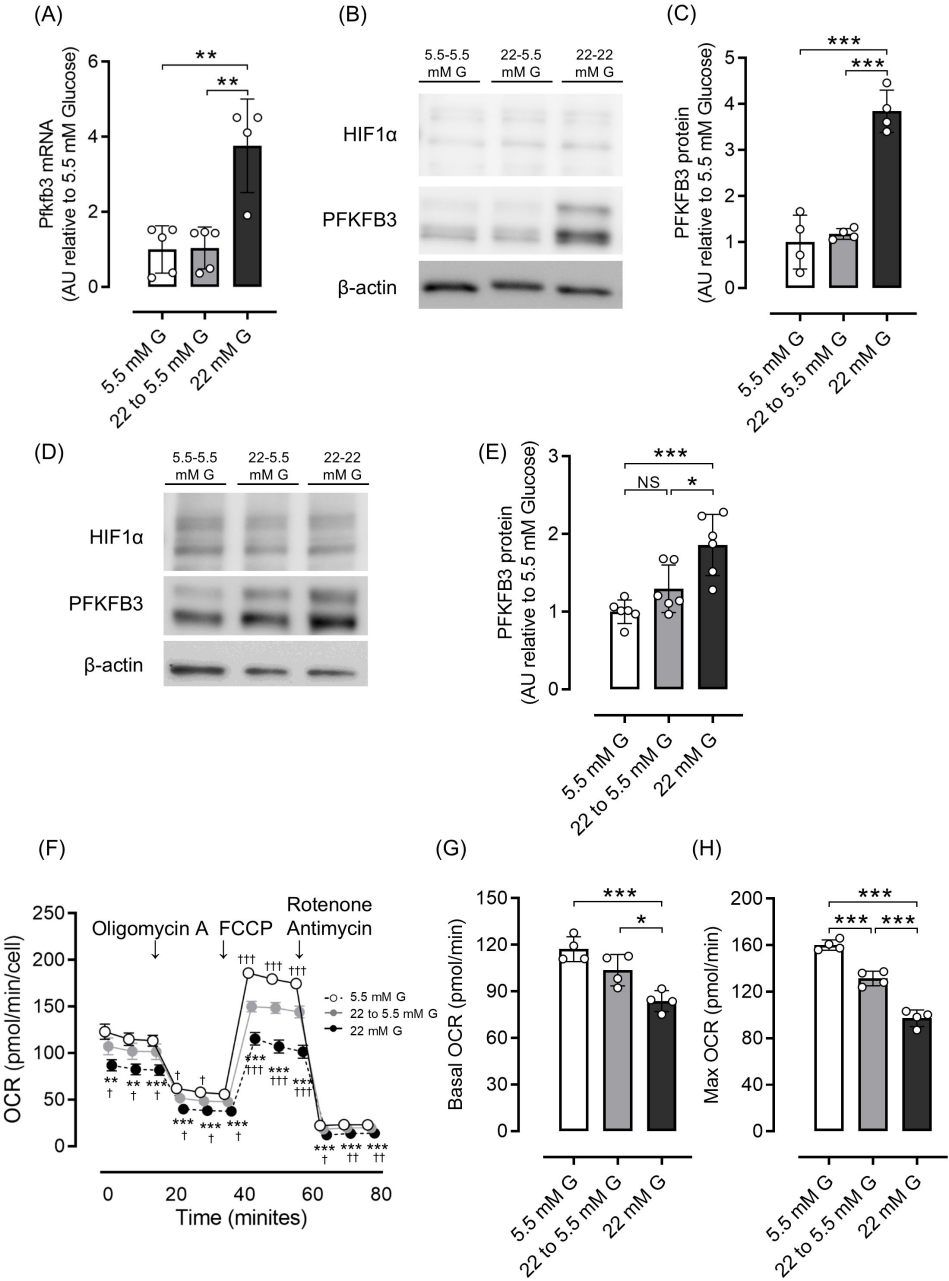
Effects of glucose concentration change on PFKFB3 expression and mitochondrial function in INS-1 832/13 cells and pancreatic islets of C57BL6/J mice. **(A)** Gene expression of *Pfkfb3* analyzed by quantitative real-time PCR using INS-1 cells collected after being cultured in low glucose (5.5 mM) or high glucose concentration (22 mM) media for 48 h, or 22 mM glucose for 24 h followed by 5.5 mM glucose for 24 h (22 to 5.5mM). Data are normalized to *beta actin* expression. **(B)** Representative Western blot of PFKFB3 and HIF1α protein levels in cell lysate from INS-1 832/13 cells cultured for 48 h in each glucose concentration. The gels and blots were cropped for clarity. For full photos, see **Supplementary Figure S11**. **(C)** Quantification of PFKFB3 protein expression in INS-1 832/13 cells under different glucose conditions. **(D)** Representative Western blot of PFKFB3 and HIF1α protein levels in pancreatic islets of C57BL6/J mice cultured under different glucose concentrations. The gels and blots were cropped for clarity. For full photos, see **Supplementary Figure S12**. **(E)** Quantification of PFKFB3 expression in islets under different glucose conditions. **(F)** Oxygen consumption rate (OCR) was measured over time (min) in INS-1 832/13 cells using an extracellular flux analyzer. Bioenergetic parameters were obtained by adding glucose (5.5, 22 to 5.5, 22 mM) to derive OCR under stimulated conditions; the ATP synthase inhibitor Oligomycin A to derive ATP-linked OCR; FCCP to uncouple the mitochondria for maximal OCR; and the mitochondrial complex inhibitor Rotenone/Antimycin A to block mitochondrial respiration. White open circles, 5.5 mM glucose; gray circles, 22 to 5.5 mM glucose; black circles, 22 mM glucose. **(G, H)** Quantification of basal OCR **(G)** and maximal OCR **(H)**. Data are mean ± SD. N = 4–5 in **(A)**, 4 in **(C)**, 6 in **(E)** and 4 in **(F–H)**. * *p <*0.05, ** *p <*0.01, *** *p <*0.001, one-way ANOVA with Bonferroni’s *post hoc* test. G, glucose; HIF1α, hypoxia inducible factor α; PFKFB3, 6-phosphofructo-2-kinase/fructose-2,6-biphosphatase 3; NS, not significant; OCR, oxygen consumption rate; FCCP, carbonyl cyanide p-trifluoro methoxyphenylhydrazone.

Next, we investigated whether the increased expression of PFKFB3 and mitochondrial dysfunction observed in a high-glucose environment could be shifted by changing glucose exposure. In both INS-1 832/13 cells and isolated islets, the elevated PFKFB3 mRNA and protein expression observed under high glucose conditions was corrected after exposure to a low-glucose environment, while the impaired mitochondrial function in INS-1 832/13 was markedly improved (**Figures 3A–H**).

**Figure 4:**
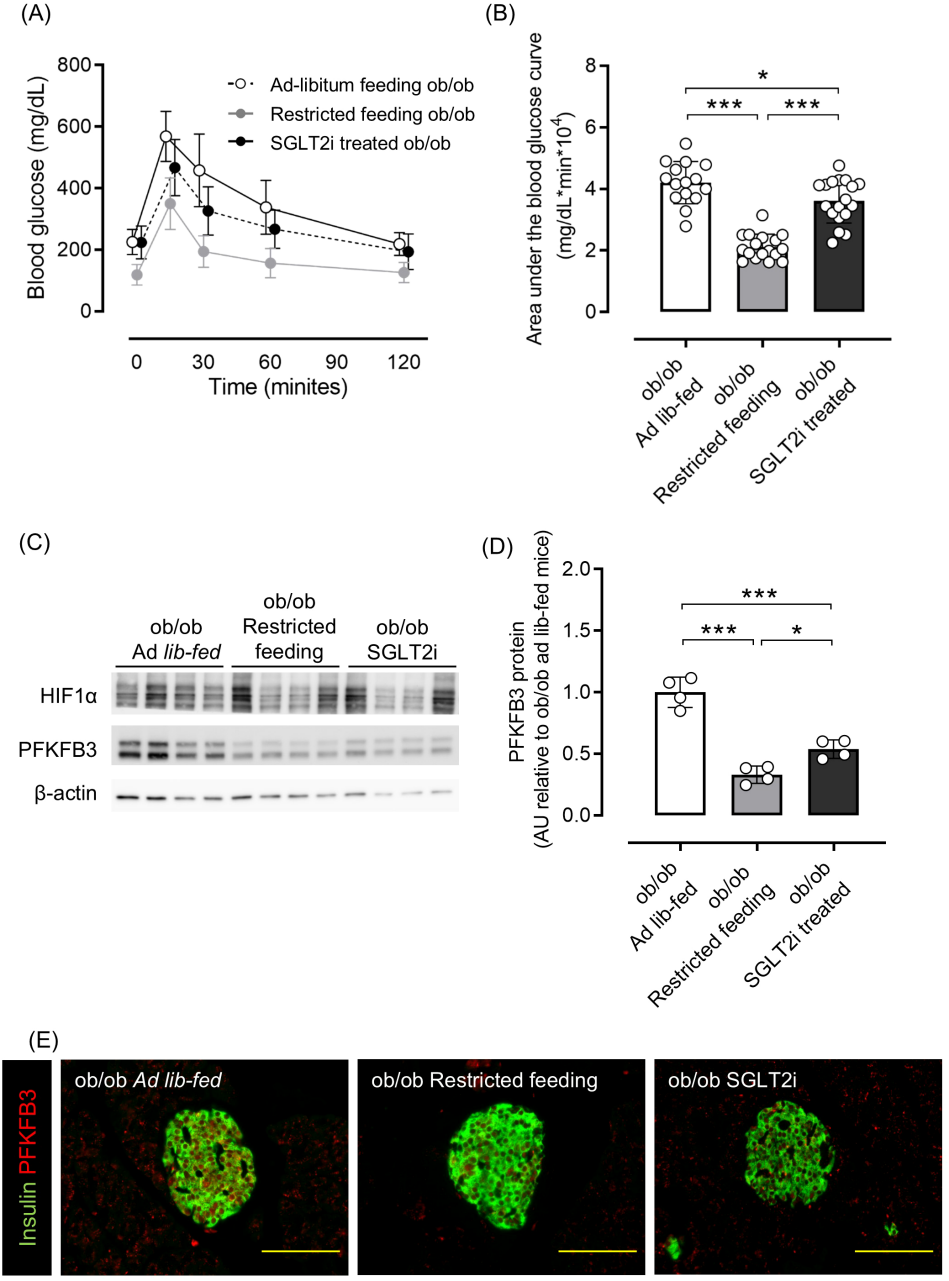
Effects of restricted feeding and SGLT2i on glucose tolerance and PFKFB3 expression in pancreatic islets of ob/ob mice. **(A)** Blood glucose changes during an oral glucose tolerance test after being fed a standard chow diet ad libitum, restricted to 2 g/day, or ad libitum with tofogliflozin added from 8 to 12 weeks of age. White circles, ad-libitum feeding ob/ob mice; gray circles, restricted feeding ob/ob mice; black circles, ad-libitum feeding, SGLT2i-treated ob/ob mice. **(B)** Area under the curve of the oral glucose tolerance test. **(C)** Representative western blot of PFKFB3 and HIF1α protein levels in pancreatic islets of ob/ob mice. The gels and blots were cropped for clarity. For full photos, see **Supplementary Figure S13**. **(D)** Quantification of PFKFB3 expression in islets under different conditions. Data are normalized to β-actin expression. White bar, ad-libitum feeding ob/ob mice; gray bar, restricted feeding ob/ob mice; black bar, ad-libitum feeding, SGLT2i-treated ob/ob mice. **(E)** Immunohistochemical staining of insulin and PFKFB3 in pancreatic islets. Red, PFKFB3; green, insulin. Yellow bar; 100 μm. Data are mean ± SD. N = 16–18 in **(A, B)** and 4 in **(D)**. * *p <*0.05, ** *p <*0.01, *** *p <*0.001, one-way ANOVA with Bonferroni’s *post hoc* test. SGLT2i, sodium-glucose cotransporter 2 inhibitor; HIF1α, hypoxia inducible factor α; PFKFB3, 6-phosphofructo-2-kinase/fructose-2,6-biphosphatase 3; AU, arbitrary units.

As shown in **Supplementary Figures 1A, B**, 8-week-old ob/ob mice were overweight and hyperglycemic compared with ob/+ mice, and PFKFB3 expression in pancreatic beta cells was already elevated. Therefore, we examined whether these metabolic changes could be corrected by two different interventions: food restriction and administration of the sodium-glucose cotransporter 2 inhibitor (SGLT2i), tofogliflozin. Tofogliflozin treatment resulted in increased food intake, slightly decreased body weight, and significantly improved glucose tolerance compared with the ad-libitum feeding group; dietary restriction resulted in a marked reduction of body weight and significantly improved glucose tolerance compared with the ad-libitum feeding group (*p <*0.001; **Supplementary Figures S4A–C**; **Figures 4A, B**). As a result of the reduction of excessive beta cell workload, the beta cell mass in the SGLT2i and food restriction groups was significantly lower than the ad-libitum feeding group in ob/ob mice (both *p <*0.05; **Supplementary Figure S5A**). Protein expression of PFKFB3 in the islets was also significantly lower in mice from these two interventions compared with the ad-libitum feeding group (both *p <*0.05; **Figures 4C–E**; **Supplementary Figure S5B**). These findings suggest that the upregulated expression of PFKFB3 under high glucose conditions and an obese-diabetic state can be flexible under low glucose conditions and inhibited by dietary and pharmacological interventions, respectively.

### The impact of PFKFB3 and glycolysis inhibition on intracellular metabolism under diabetic conditions

**Figure 5:**
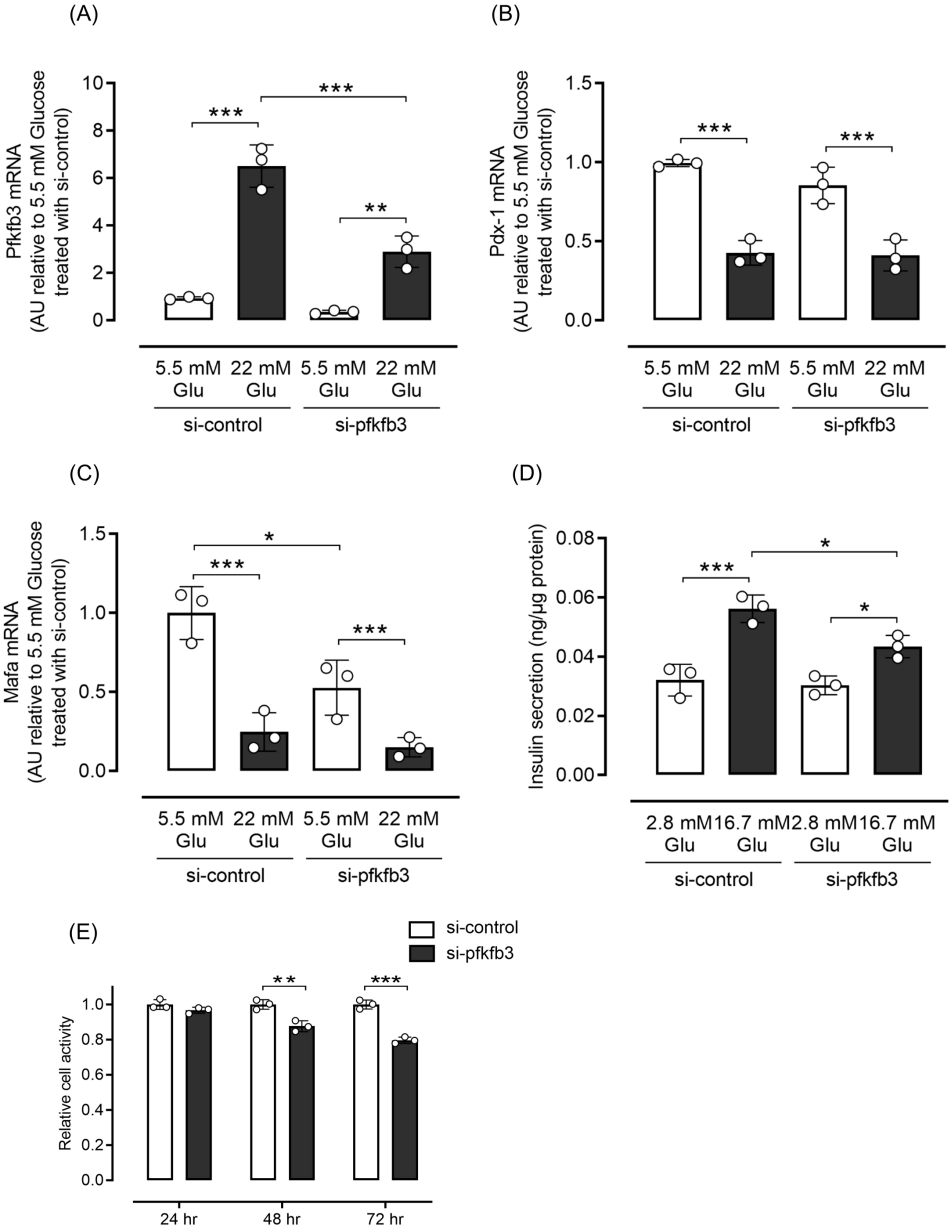
Effects of silencing *Pfkfb3* expression in INS-1 832/13 cells. **(A)** Gene expression of *Pfkfb3*, **(B)** *Pdx1* and **(C)** *Mafa* analyzed by quantitative real-time PCR using INS-1 cells collected after being cultured for 48 h with the administration of siRNA against *Pfkfb3* or control siRNA under either 5.5 or 22 mM glucose conditions. Data are normalized by *beta actin* expression. **(D)** Insulin secretion during the glucose tolerance test. **(E)** Cell viability was assessed by MTT assays in INS-1 832/13 cells transfected with *Pfkfb3* siRNA or control siRNA and cultured under 11 mM glucose conditions for 24, 48, or 72 h. Absorbance at 570 nm was measured and normalized to the control siRNA group (siCONT) at each time point. MTT measurements were performed in technical triplicate. A time-dependent decrease in cellular metabolic activity was observed in cells with *Pfkfb3* knockdown, which reached statistical significance at 48 and 72 h. Data are mean ± SD. N = 3 in each condition **(A–E)**. * p <0.05, ** p <0.01, *** p <0.001, one-way ANOVA with Bonferroni’s *post hoc* test. Glu, glucose; PFKFB3, 6-phosphofructo-2-kinase/fructose-2,6-biphosphatase 3; Pdx-1, pancreatic and duodenal homeobox 1; Mafa, v-maf musculoaponeurotic fibrosarcoma oncogene homolog A; AU, arbitrary units; MTT, 3-(4,5-dimethylthiazol-2-yl)-2,5-diphenyltetrazolium bromide.

Considering that the expression of PFKFB3 in pancreatic beta cells was upregulated under obese hyperglycemic conditions, and is known to affect intracellular metabolic changes, we next examined whether interventions modulating PFKFB3 expression affected the fate of pancreatic beta cells. Expression of *Pdx1* and *Mafa*, transcriptional factors important for pancreatic beta cell function, was significantly lower in INS-1 cells cultured in high-glucose medium than in low-glucose medium (both *p <*0.001; **Figures 5B, C**). Silencing of the *Pfkfb3* gene using siRNA did not rescue the expression of transcriptional factors (**Figures 5A–C**). However, glucose-stimulated insulin secretion was significantly lower under high-glucose stimuli in the group under *Pfkfb3* silencing than in the high-glucose si-control group (*p <*0.05; **Figure 5D**). To further investigate the functional impact of *Pfkfb3* knockdown, we evaluated cellular metabolic activity using an MTT assay in INS-1 832/13 cells cultured with 11 mM glucose. While no significant difference in MTT absorbance was observed at 24 hours post-transfection, absorbance was significantly lower in cells treated with Pfkfb3 siRNA than those treated with control siRNA at 48 hours (p < 0.01), an effect that became more pronounced at 72 hours (p < 0.001).

These findings indicate that PFKFB3 suppression impairs cellular metabolic activity in a time-dependent manner, which may result from reduced beta cell viability and/or proliferative capacity under physiological glucose conditions.

**Figure 6:**
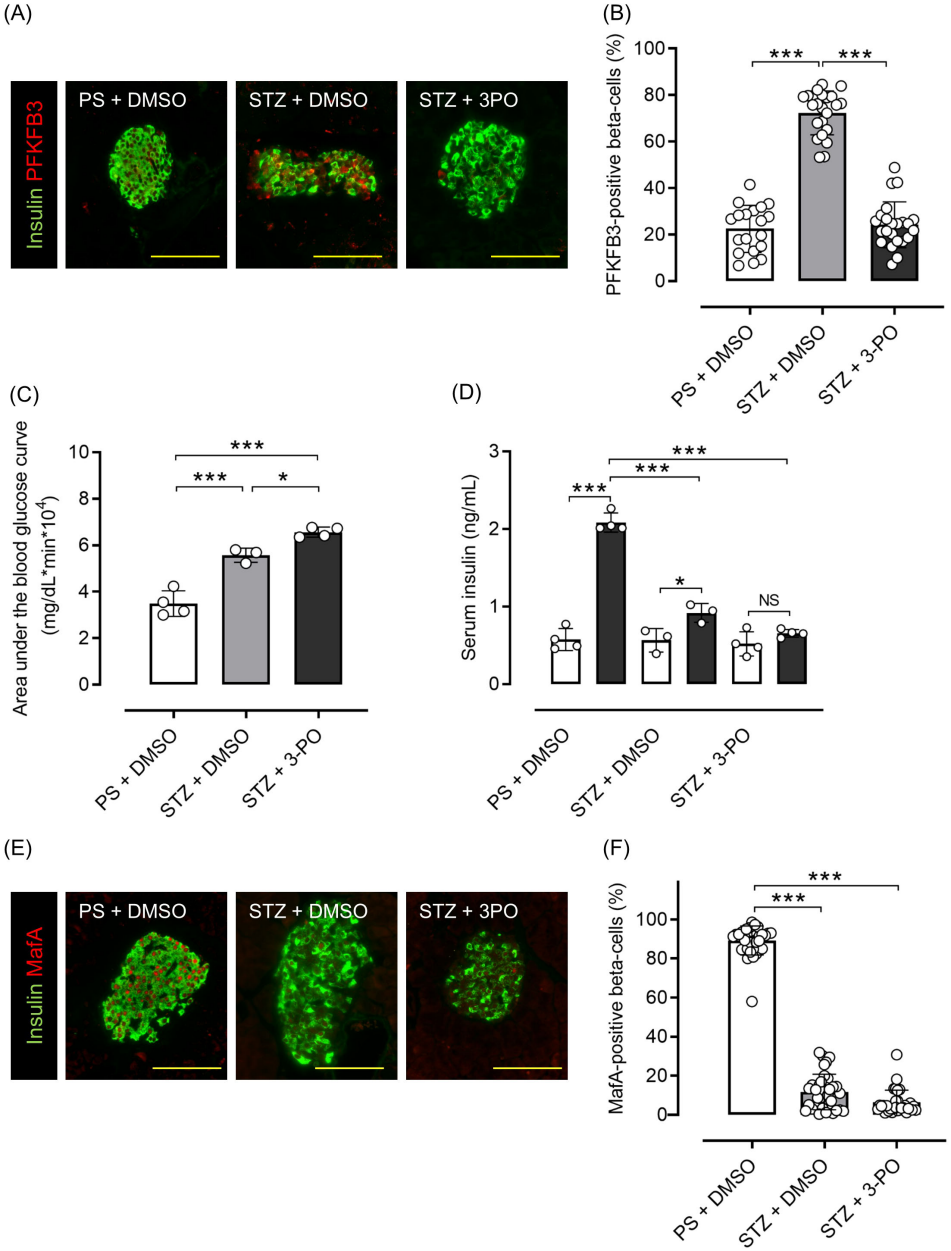
Effects of glycolysis inhibition and PFKFB3 modulation on glucose tolerance, gene expression in pancreatic islets and insulin secretion in C57BL6/J mice treated with or without streptozotocin (STZ). **(A)** Immunohistochemical staining of insulin and PFKFB3 in pancreatic islets of C57B6/J mice treated in three different ways: the PS + DMSO group (control) received saline for 5 days followed by DMSO every other day for 2 weeks, the STZ + DMSO group received STZ (50 mg/kg/day for 5 days) followed by DMSO every other day for 2 weeks, and the STZ + 3PO (glycolysis inhibitor) group received STZ (50 mg/kg/day for 5 days) followed by 3PO (50 μg/g/day every other day for 2 weeks). Green, insulin; red, PFKFB3. **(B)** Percentage of PFKFB3-positive beta cells in immunohistochemical staining of pancreatic islets. **(C)** Area under the curve of the oral glucose tolerance test. **(D)** Insulin levels during the glucose tolerance test. White bars, insulin levels at 0 min; black bars, insulin levels at 15 min. **(E)** Immunohistochemical staining of insulin and MafA in pancreatic islets. Green, insulin; red, MafA. **(F)** Percentage of MafA-positive beta cells in immunohistochemical staining of pancreatic islets. Data are mean ± SD. N = 19–23 isolated pancreatic islets from three mice in each group in **(B)**, 3–4 mice in **(C, D)**, 30–35 isolated pancreatic islets from three mice in each group in **(F)**. * *p <*0.05, *** *p <*0.001, one-way ANOVA with Bonferroni’s *post hoc* test. STZ, streptozotocin; 3PO, 3-(3-pyridinyl)-1-(4-pyridinyl)-2-propen-1-one; DMSO, dimethyl sulfoxide; PS, physiological saline; PFKFB3, 6-phosphofructo-2-kinase/fructose-2,6-biphosphatase 3; MafA, v-maf musculoaponeurotic fibrosarcoma oncogene homolog A. ns, not significant.

Mice with streptozotocin (STZ)-induced diabetes had significantly higher PFKFB3 expression in pancreatic beta cells than control-treated mice (*p <*0.001), which was significantly inhibited by 3-(3-pyridinyl)-1-(4-pyridinyl)-2-propen-1-one (3PO), a glycolysis inhibitor (*p <*0.001; **Figures 6A, B**). Although body weight was not different between the 3PO-treated group and the control group, there was an increase in blood glucose levels in the 3PO-treated group compared to the control group throughout the study (**Supplementary Figures S6A, B**). In the OGTT, the 3PO-treated group had significantly lower glucose tolerance and insulin secretion compared with the control group (both *p <*0.001; **Figures 6C, D**; **Supplementary Figure S6C**). MafA expression in islets was also significantly lower after STZ administration, and 3PO treatment did not correct this change (both *p <*0.001 vs. control; **Figures 6E, F**). Additionally, pancreatic beta cell mass was significantly lower after STZ administration (by 88%), which was not altered by 3PO (both *p <*0.001 vs. control; **Supplementary Figure S7**). Based on both cell and mouse experiments, results suggest that knockdown of PFKFB3 or pharmacological inhibition of glycolysis, both of which impair a pathway that is upregulated under diabetic conditions, worsens glucose tolerance.

## Discussion

This study investigated the role of the glycolytic pathway in the metabolic adaptation of rodent pancreatic beta cells under acute metabolic stress. Using *in vitro* and *in vivo* models, including INS-1 832/13 cells and STZ-treated mice, we examined whether modulation of the glycolytic pathway via its key regulator, PFKFB3, influenced insulin secretion and glucose tolerance. Our results indicate that upregulation of PFKFB3 under acute metabolic stress may function as a short-term, compensatory mechanism to maintain beta cell viability and support insulin output, but this benefit appears to be context- and time-dependent.

PFKFB3 expression was increased under an excessive metabolic workload, consistent with previous reports showing upregulation of glycolytic enzymes in the islets of Goto Kakizaki rats and beta cell lines exposed to high glucose concentrations (16–18), with changes at both the mRNA and protein levels (19). Moreover, PFKFB3 is overexpressed in pancreatic islets from individuals with type 2 diabetes (12, 20). Importantly, our data demonstrated that this upregulation of PFKFB3 could be reversed by reducing metabolic stress via dietary or pharmacological interventions. Suppression of the glycolytic pathway, either by siRNA-mediated knockdown or 3PO-mediated pharmacological inhibition of glycolysis, resulted in modest but significant reductions in insulin secretion and glucose tolerance. These observations, while supporting a transient beneficial effect of PFKFB3 activity during acute metabolic overload, must be interpreted within the limitations of short-term experimental conditions and may not fully represent chronic diabetic states.

Distinguishing between acute and chronic stress is essential when interpreting the role of PFKFB3. Although increased PFKFB3 expression has been observed in pancreatic islets from individuals with long-standing diabetes (12, 20), the functional significance of this upregulation may vary over time. In early adaptive phases, PFKFB3 upregulation may help preserve beta cell function. However, persistent or chronic upregulation may become maladaptive and contribute to beta cell dysfunction, as shown in chronic models (21, 22). Our findings thus support a temporally dynamic, context-dependent role for PFKFB3 beneficial in short term, potentially harmful in the long term.

In INS-1 832/13 cells, which are highly proliferative and metabolically flexible, *Pfkfb3* knockdown led to a reduction in insulin secretion, although glucose responsiveness (stimulation index) was preserved. This observation aligns with the role of glycolysis in ATP generation and insulin secretion (6, 23). Furthermore, MTT assays revealed a time-dependent reduction in cellular metabolic activity following *Pfkfb3* knockdown, most likely indicating compromised cell viability under acute stress conditions (**Figure 5E**). Recent findings by Raval et al. (21) support the view that PFKFB3-positive beta cells adopt an energy-conserving, proteostatically adapted phenotype that promotes survival under stress. These cells reduce biosynthetic and mitochondrial activity, which may indirectly limit proliferative drive, allowing time for injury repair before functional recovery. This aligns with our observation that suppression of PFKFB3 under acute stress conditions reduced cellular metabolic activity and impaired insulin secretion, reinforcing the hypothesis that short-term upregulation of PFKFB3 supports beta cell survival as a prerequisite for function. PFKFB3 localizes to both the cytoplasm and nucleus, where it catalyzes the synthesis of nuclear fructose-2,6-bisphosphate and increases the expression of key cell cycle regulators, including cyclin D3 and cell division cycle 25C (24). PFKFB3 also participates in Ras signaling, a pathway crucial in glucose metabolism and oncogenesis (25), and is strongly regulated by HIF1α under hypoxic conditions (9, 26–28), a feature observed in multiple cancers (29–32). Furthermore, modulation of the glycolytic pathway by 3PO induces apoptosis in cancer cells (33), which supports PFKFB3’s role in cell survival and stress adaptation.

Our *in vivo* studies using ob/ob mice, a model of nutritional overload, further support the reversibility and adaptability of PFKFB3 regulation under metabolic stress. Although these mice do not fully replicate human type 2 diabetes, they provide useful insights into beta cell adaptation to sustained metabolic challenges. The observed variability in PFKFB3 levels among animals and interventions underscores the complexity of its regulation *in vivo*. The STZ-induced diabetes model, which results in near-total beta cell loss, was used to assess residual metabolic responses under severe diabetic stress. After 3PO treatment, the ratio of PFKFB3-positive to negative cells appeared to be reduced. Given that 3PO reduces fructose-2,6-bisphosphate levels but does not directly bind to PFKFB3 (34), this effect is likely secondary to changes in cell viability and/or metabolic flux, rather than direct inhibition of PFKFB3 expression. Indeed, 3PO has several limitations as a glycolytic inhibitor, including its modest specificity and off-target effects. To better clarify the role of PFKFB3 in beta cell metabolism, future studies should incorporate more selective inhibitors, such as AZ67, as well as genetic approaches, including beta cell-specific *Pfkfb3*-knockout models. These tools will be essential for accurately defining the physiological and pathological functions of PFKFB3 under both acute and chronic metabolic stress.

Although increased PFKFB3-mediated glycolysis appears to compensate for mitochondrial dysfunction to promote glucose-induced insulin secretion, this finding should be treated with caution. While some studies suggest that glycolytic ATP can support insulin secretion independently of mitochondrial metabolism, prior work has shown that HIF1α-driven metabolic remodeling can lead to the replacement of glucokinase with hexokinase, a shift that favors basal rather than glucose-stimulated insulin secretion (12). Thus, the increased insulin secretion we observed in association with PFKFB3 upregulation may reflect enhanced basal insulin output rather than improved glucose responsiveness.

This study has several limitations. First, each experimental model introduces specific confounders. INS-1 832/13 cells are tumor-derived and highly proliferative, which limits their relevance to mature human beta cells. The ob/ob mouse model is based on a rare leptin mutation and exhibits an extreme metabolic phenotype. STZ induces acute beta cell destruction rather than the chronic progression of beta cell dysfunction seen in human type 2 diabetes. Second, we did not evaluate the effects of PFKFB3 overexpression, which would have provided a more comprehensive understanding of its function. Third, the use of 3PO as a glycolysis inhibitor is limited by its lack of specificity, and we did not assess alternative inhibitors such as AZ67. Fourth, we did not examine the effects of PFKFB3 modulation on apoptosis, cell cycle progression, or alternative metabolic pathways such as the pentose phosphate pathway or lipid metabolism. Fifth, immunofluorescence in ob/ob mice showed relatively strong PFKFB3 signals in exocrine pancreas tissue (**Figures 4E**), and background fluorescence in endocrine compartments complicated image interpretation. Split-channel images are provided in the **Supplementary Materials** for transparency. Lastly, although HIF1α and PFKFB3 were both elevated in islets under metabolic overload, we did not observe a consistent correlation between their expression levels, which suggests that additional regulatory factors may be involved. This discrepancy implies that while HIF1α may contribute to the initial transcriptional activation of PFKFB3, its expression alone is not a reliable predictor of PFKFB3 protein abundance or functional engagement in beta cells under stress. Other glucose-sensitive or stress-responsive pathways, such as AMP-activated protein kinase or mammalian target of rapamycin signaling, may also play a regulatory role and warrant further investigation.

In summary, our study supports a model in which PFKFB3 upregulation confers a short-term adaptive survival advantage to beta cells during acute metabolic stress, but persistent activation may carry long-term risks. The temporally dynamic, context-dependent role of PFKFB3 should be carefully considered when targeting metabolic pathways for diabetes therapy. However, our data also indicate that this upregulation primarily functions to promote beta cell survival under stress conditions, with effects on insulin output potentially occurring as a secondary consequence. These findings support the idea that PFKFB3 upregulation functions as a short-term adaptive mechanism under metabolic pressure. However, considering its potential maladaptive role in chronic stress, further studies using chronic disease models, genetic manipulation, and more selective pharmacological tools are necessary. Our work highlights the temporally dynamic role of PFKFB3 in beta cell physiology and underscores the importance of context when targeting metabolic pathways for diabetes therapy.

## Data Availability

The original contributions presented in the study are included in the article/**Supplementary Material**. Further inquiries can be directed to the corresponding author.
