# Supplementary material for: Metabolic adaptation to acute metabolic stress via PFKFB3 upregulation in rodent beta cells
Source: Front Endocrinol (Lausanne). 2025 Jun 17;16:1552700. doi: 10.3389/fendo.2025.1552700 (PMC12208841; doi:10.3389/fendo.2025.1552700)
Supplement: Supplementary file 1 [file DataSheet1.doc]

**Supplementary Methods**

Influence of glucose stimulation on metabolic changes:
1-1) INS-1 832/13 cells and isolated pancreatic islets from male C57BL/6J mice were cultured with RPMI medium containing 10% FBS at 5.5, 11 and 22 mM glucose environment for 48 hours. Gene and protein expression changes were analyzed, and mitochondrial function was evaluated using an extracellular flux analyzer.

1-2) Male ob/+ mice and ob/ob mice were fed a standard chow freely with powdered CE-2 diet until 6-10 weeks of age. Additionally, a group with CE-2 diet intake restricted to 2 g/day (Restricted intake group) was established. Body weight, blood glucose level and glucose tolerance (OGTT) were compared among the groups. At 10 weeks of age, immunostaining of pancreatic tissue and Western Blot analysis of isolated pancreatic islets were performed.

Flexibility of intracellular metabolic changes:
2-1) INS-1 832/13 cells and islets of C57BL/6J mice were cultured at low glucose (5.5 mM: low G), high glucose concentration (22 mM: high G) for 48 hours or high G for 24 hours followed by low G for another 24 hours. Changes in gene and protein expression and mitochondrial function were analyzed by Western blotting and extracellular flux analyzer.

2-2) Eight-week-old ob/ob mice were fed a standard chow ad lib or the same diet restricted to 2 g/day, or tofogliflozin added chow ad lib for 4 weeks. The body mass and glucose tolerance were compared among the groups. In addition, glucose tolerance was evaluated using OGTT. At 12 weeks of age, immunostaining of pancreatic tissue, Western blotting method and DNA microarray analysis of isolated pancreatic islets were performed.

Intervention on intracellular metabolic changes:
3-1) INS-1 832/13 cells were cultured for 48 hours with the administration of PFKFB3 siRNA, separately, under either low glucose or high glucose conditions. Gene expression and glucose-stimulated insulin secretion were compared.
3-2) As an inhibitor of glycolytic pathway, 3-(3-pyridinyl)-1-(4-pyridinyl)-2-propen-1-one (3PO) was used. Male C57B/6J mice aged 16 to 20 weeks were treated in three different ways: phosphate buffered saline + DMSO group as control group received saline for 5 days followed by DMSO every other day for 2 weeks, streptozotocin (STZ) + DMSO group received STZ (50 mg/kg/day for 5 days) followed by DMSO every other day for 2 weeks, and STZ + 3PO (glycolysis inhibitor) group received STZ (50 mg/kg/day for 5 days) followed by 3PO (50 μg/g/day every other day for 2 weeks). Glucose tolerance was evaluated using oral glucose tolerance test, and MafA expression in pancreatic islets was evaluated using immunostaining.

**Supplementary Figures**


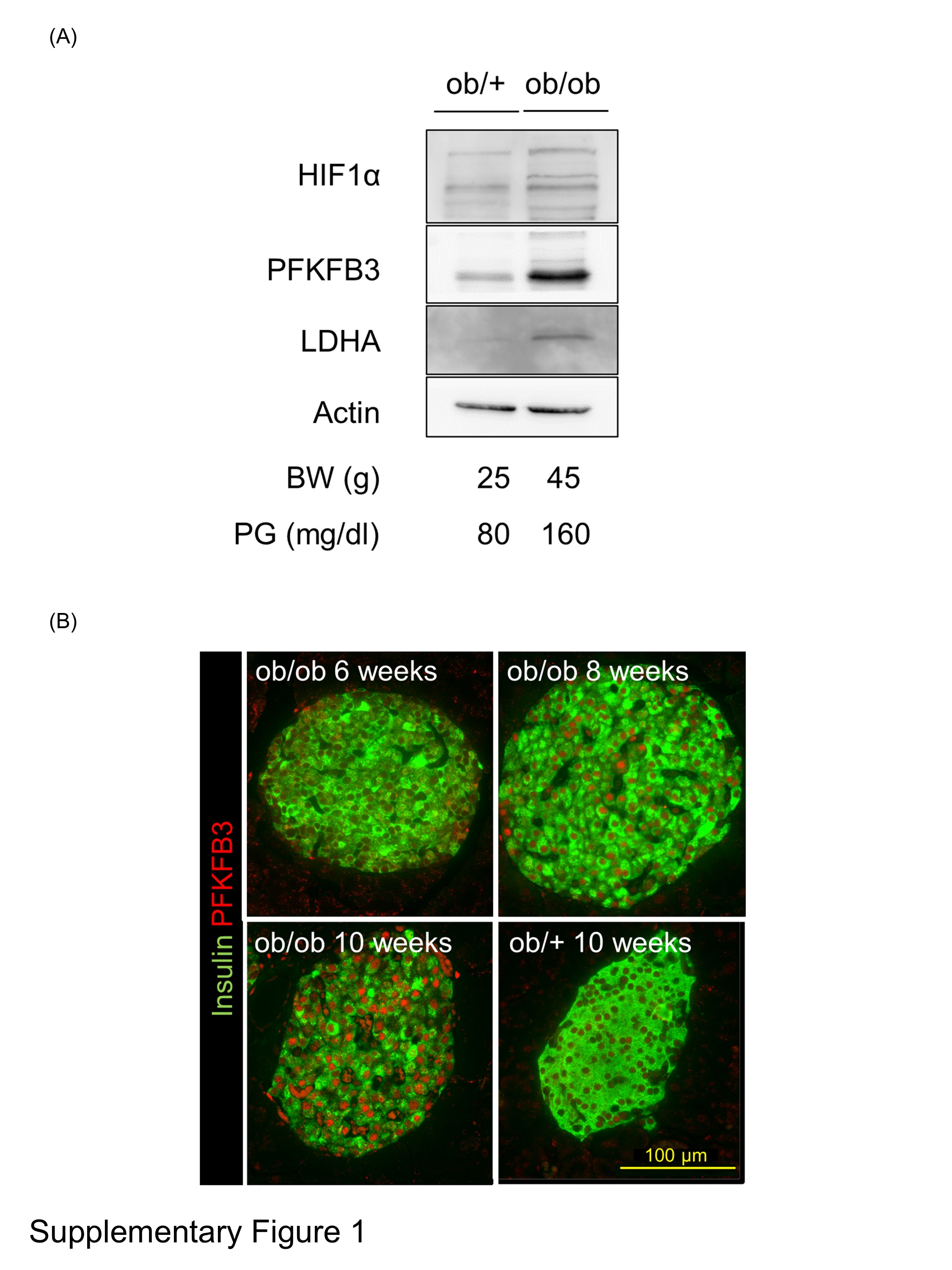


**Supplementary Figure S1. PFKFB3 expression in pancreatic islets of ob/+ and ob/ob mice.**

(A) Representative Western blot of PFKFB3 and HIF1α protein levels in pancreatic islets of ob/+ and ob/ob mice. (B) Immunohistochemical staining of insulin and PFKFB3 in pancreatic islets. Red, PFKFB3; green, insulin. HIF1α, hypoxia inducible factor α; PFKFB3, 6-phosphofructo-2-kinase/fructose-2,6-biphosphatase 3; LDHA, lactate dehydrogenase A; BW, body weight; PG, plasma glucose.


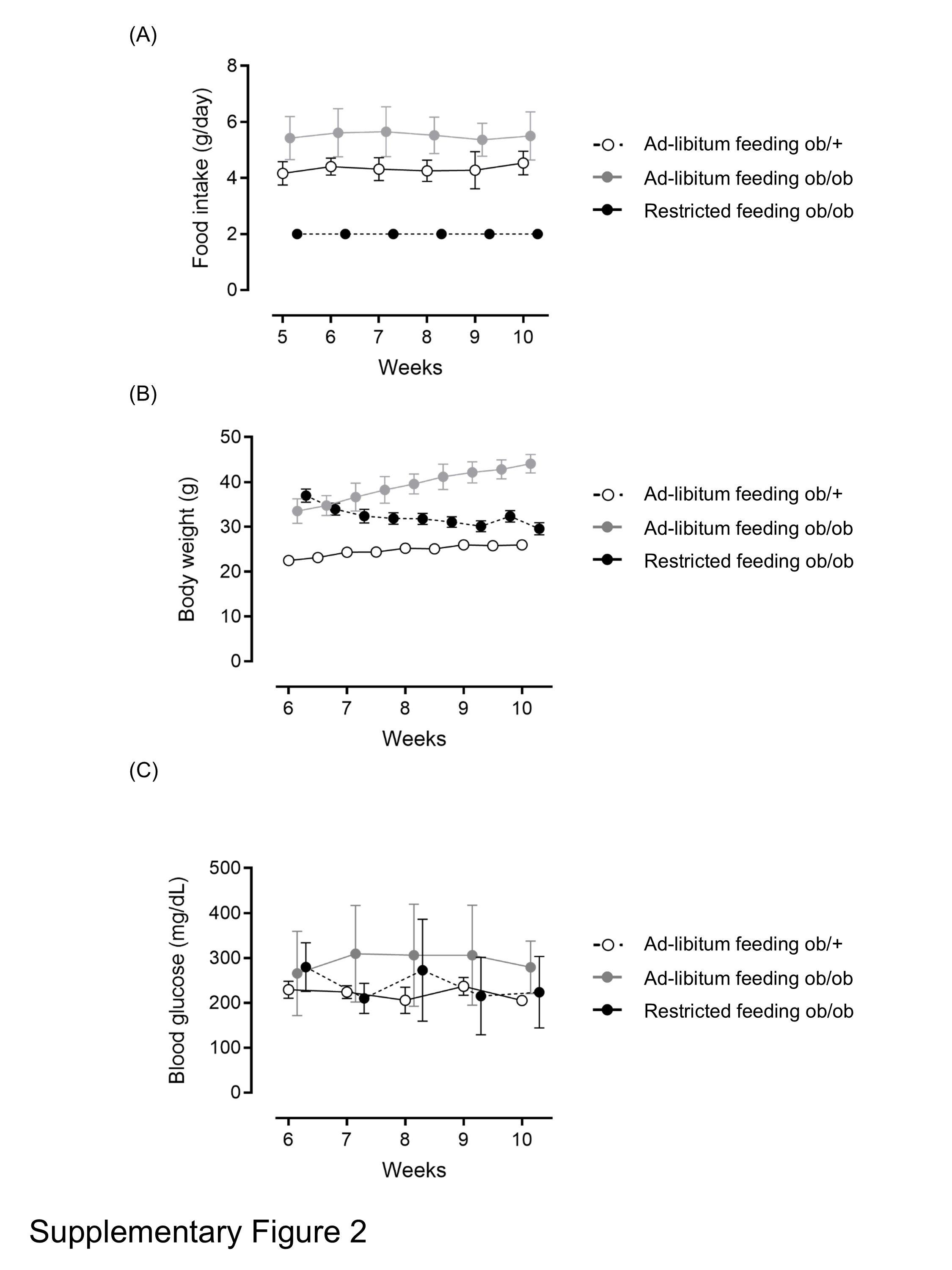


**Supplementary Figure S2. Effects of restricted feeding on body weight and blood glucose of ob/ob mice.**

(A) Food intake, (B) body weight, and (C) blood glucose changes after being fed a standard chow diet ad libitum or restricted to 2 g/day from 6 to 10 weeks of age. White circles, ad-libitum feeding ob/+ mice; gray circles, ad-libitum feeding ob/ob mice; black circles, restricted feeding ob/ob mice. Data are the mean ± SD. N = 6–10 in (A, B, C).


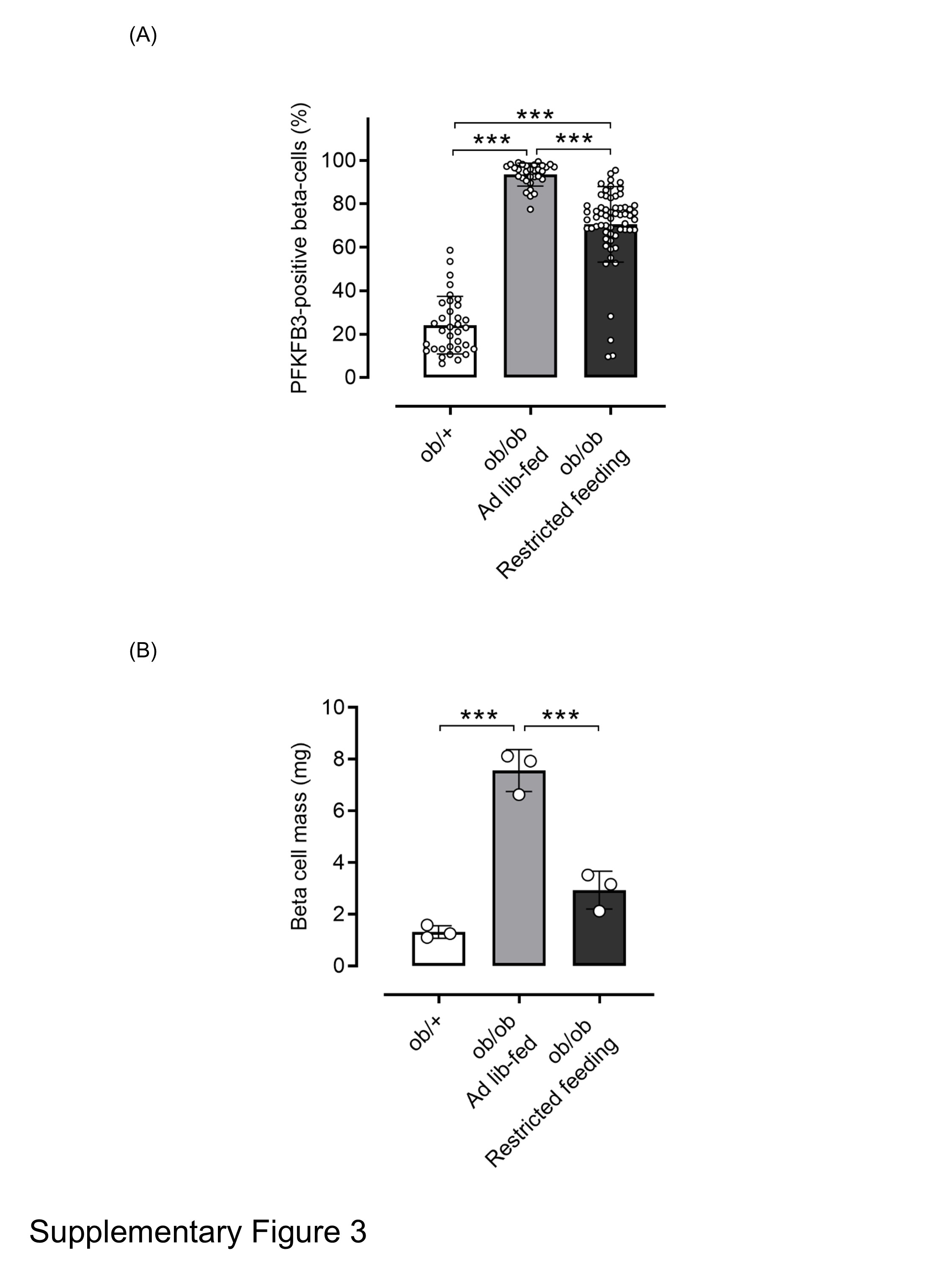


**Supplementary Figure S3. Immunohistochemical staining of ad-libitum feeding ob/+ mice, ad-libitum feeding ob/ob mice and restricted feeding ob/ob mice.**

(A) PFKFB3-positive beta cells, (B) beta cell mass of ad-libitum feeding ob/+ mice, ad-libitum feeding ob/ob mice and restricted feeding ob/ob mice. White bar, ad-libitum feeding ob/+ mice; gray bar, ad-libitum feeding ob/ob mice; black bar, restricted feeding ob/ob mice. Data are mean ± SD. N = 31–61 pancreatic islets from three mice in each group in (A), 3 mice in (B). * *p* <0.05, ** *p* <0.01, *** *p* <0.001, one-way ANOVA with Bonferroni’s post hoc test. Beta cell mass was calculated as follows: beta cell mass (mg) = (beta cell area ∕ pancreatic area) × pancreas weight (mg).


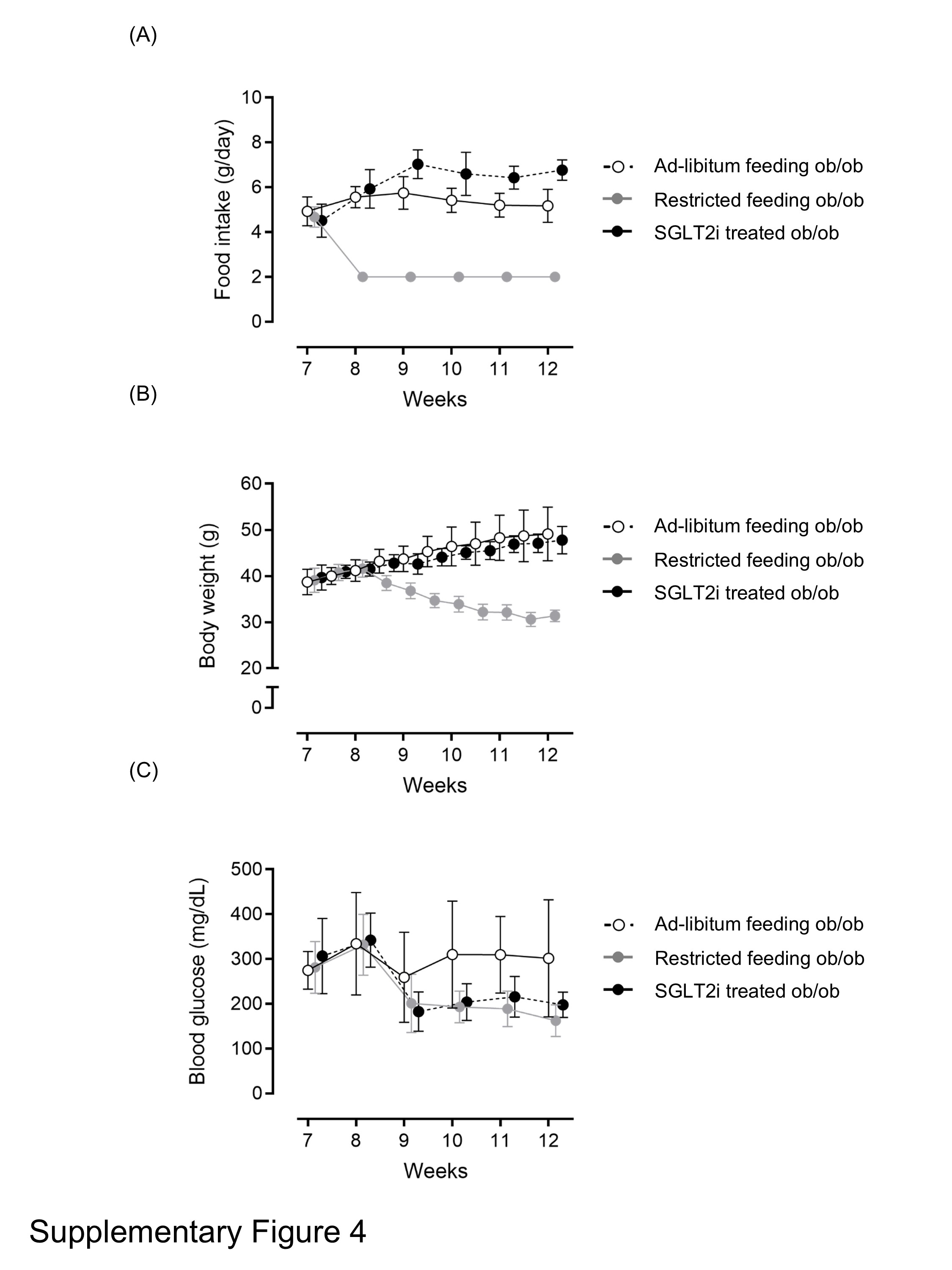


**Supplementary Figure S4. Effects of restricted feeding and SGLT2i on body weight and blood glucose of ob/ob mice.**

(A) Food intake, (B) body weight, and (C) blood glucose changes in ob/ob mice after being fed a standard chow diet ad libitum, restricted to 2 g/day, or ad libitum with tofogliflozin added from 8 to 12 weeks of age. White circles, ad-libitum feeding ob/ob mice; gray circles, restricted feeding ob/ob mice; black circles, ad-libitum feeding, SGLT2i-treated ob/ob mice. SGLT2i, sodium-glucose cotransporter 2 inhibitor. Data are the mean ± SD. N = 16–18 in (A, B, C).


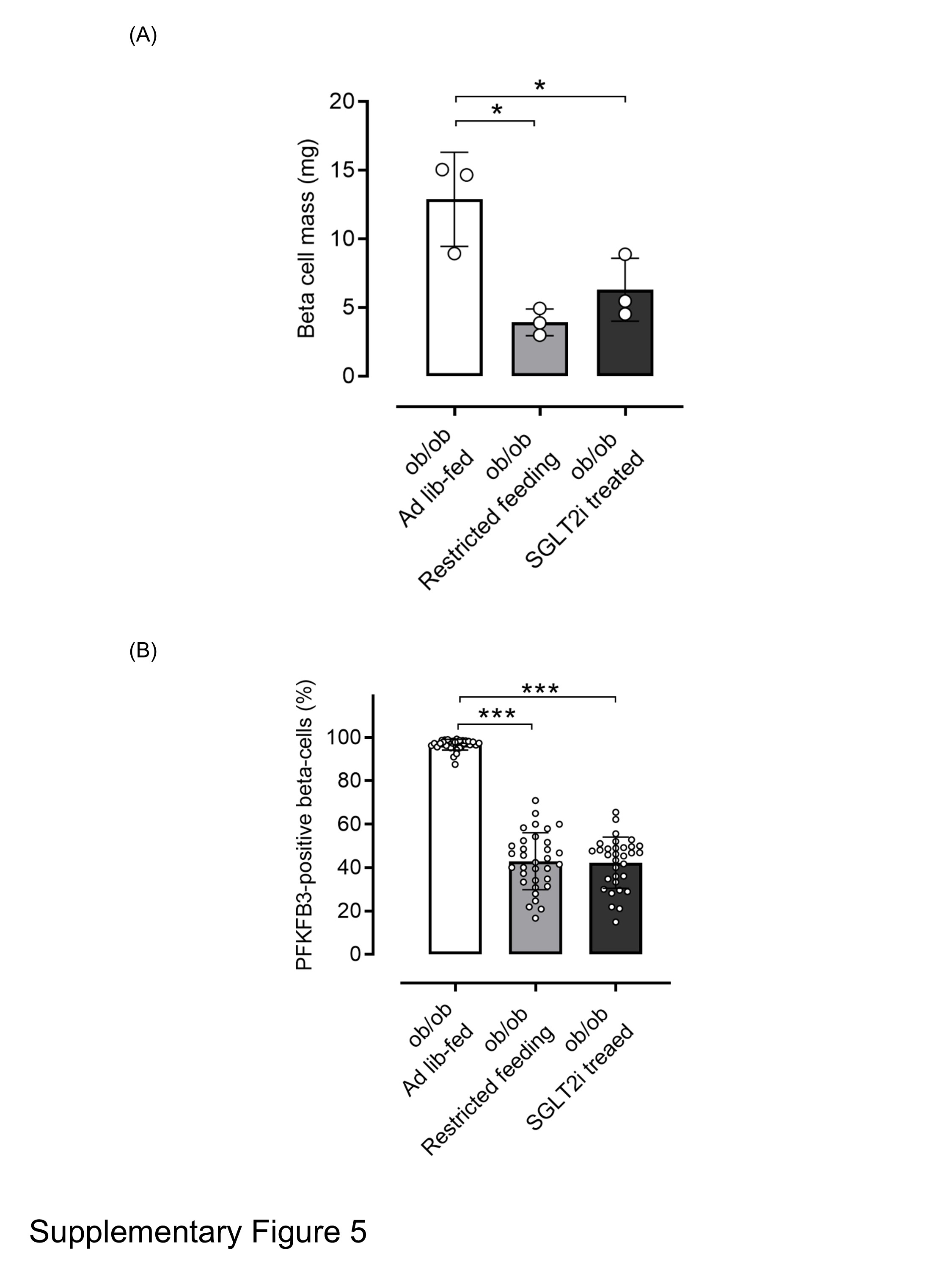


**Supplementary Figure S5. Immunohistochemical staining of ad-libitum feeding ob/ob mice, restricted feeding ob/ob mice and ad-libitum feeding, SGLT2i-treated ob/ob mice.**

(A) Beta cell mass and (B) PFKFB3-positive beta cells from ad-libitum feeding ob/ob mice, restricted feeding ob/ob mice and ad-libitum feeding, SGLT2i-treated ob/ob mice. White bar, ad-libitum feeding ob/ob mice; gray bar, restricted feeding ob/ob mice; black bar, ad-libitum feeding, SGLT2i-treated ob/ob mice. Data are mean ± SD. N = 3 mice in (A), N = 32–34 isolated pancreatic islets from three mice in each group in (b). * *p* <0.05, ** *p* <0.01, *** *p* <0.001, one-way ANOVA with Bonferroni’s post hoc test. Beta cell mass was calculated as follows: beta cell mass (mg) = (beta cell area ∕ pancreatic area) × pancreas weight (mg). SGLT2i, sodium-glucose cotransporter 2 inhibitor.


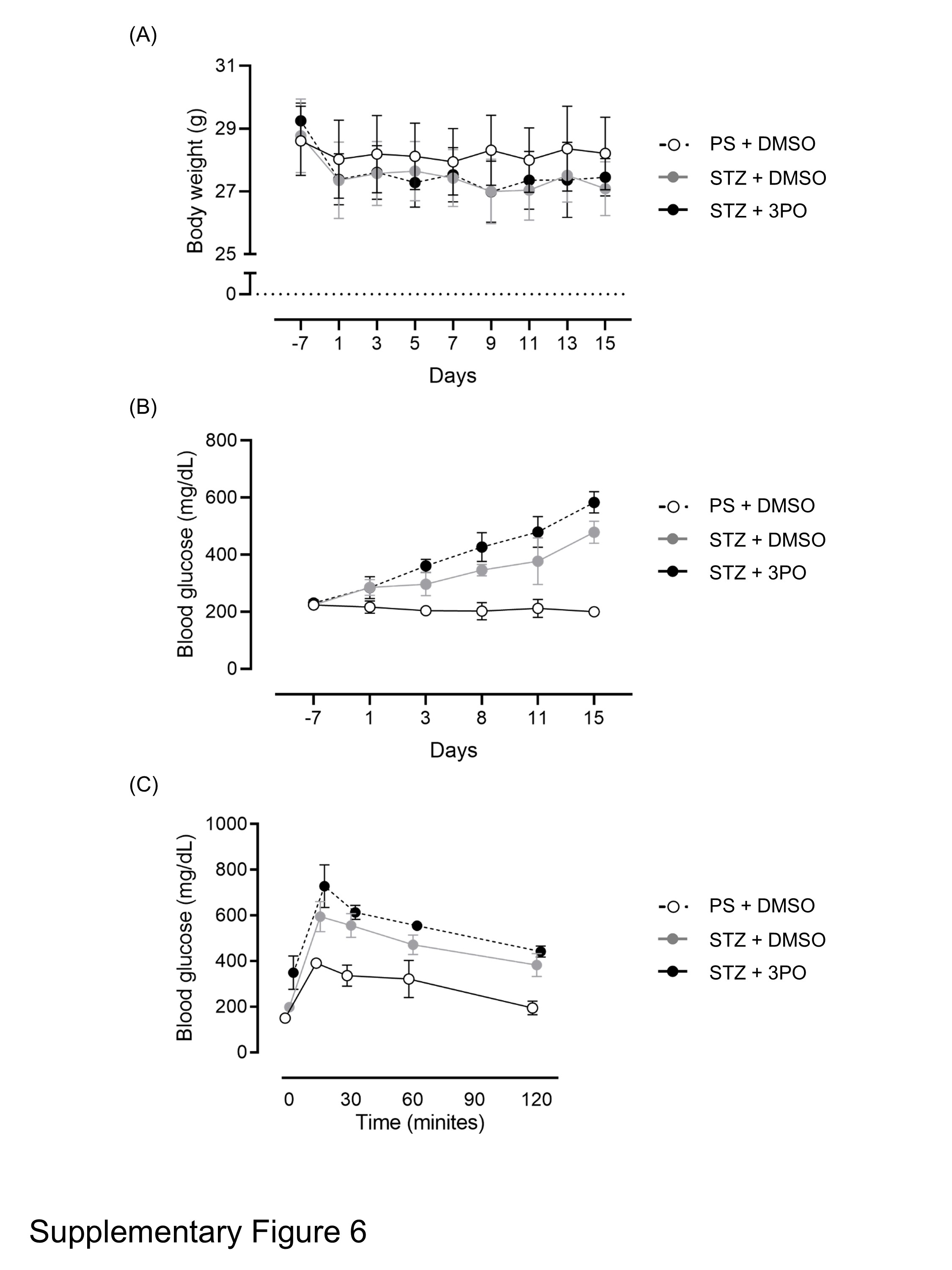


**Supplementary Figure S6. Effects of inhibiting glycolysis on body weight and blood glucose of C57BL6/J mice treated with or without streptozotocin.**

(A) Body weight, (B) Blood glucose, and (C) blood glucose changes after an oral glucose tolerance test in C57B6/J mice after being treated in three different ways: the PS + DMSO group (control) received saline for 5 days followed by DMSO every other day for 2 weeks, the STZ + DMSO group received streptozotocin (STZ) (50 mg/kg/day for 5 days) followed by DMSO every other day for 2 weeks, and the STZ + 3PO (glycolysis inhibitor) group received STZ (50 mg/kg/day for 5 days) followed by 3PO (50 μg/g/day every other day for 2 weeks). White circles, PS + DMSO group; gray circles, STZ + DMSO group; black circles, STZ + 3PO group. Data are mean ± SD. N = 3–4 in (A, B, C). PS, physiological saline; DMSO, dimethyl sulfoxide; STZ, streptozotocin; 3PO, 3-(3-pyridinyl)-1-(4-pyridinyl)-2-propen-1-one.


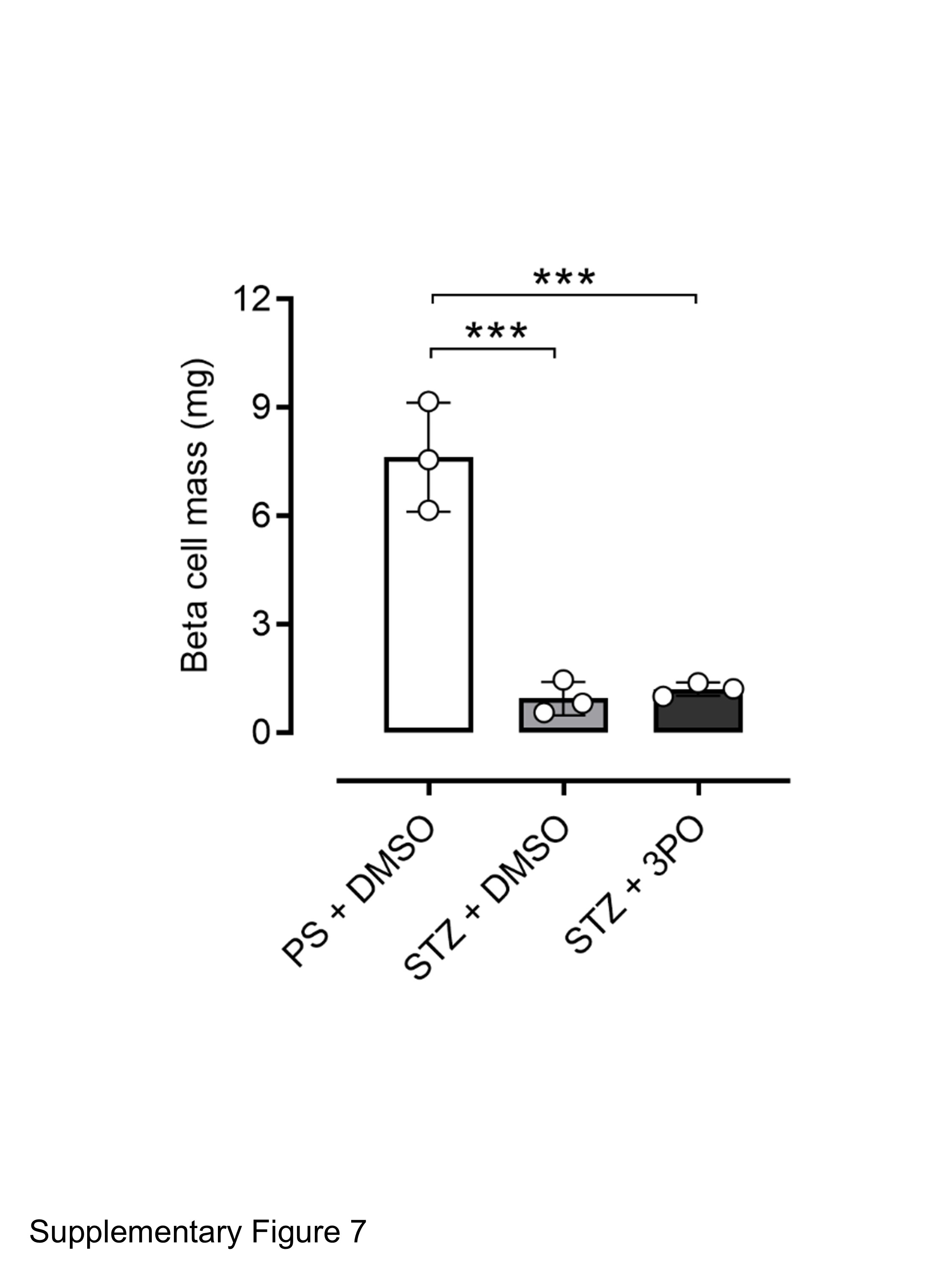


**Supplementary Figure S7. Effects of inhibiting glycolysis on beta cell mass of C57BL6/J mice treated with or without streptozotocin.**

Beta cell mass of the PS + DMSO C57B6/J mouse group, the STZ + DMSO C57B6/J mouse group and the STZ + 3PO C57B6/J mouse group. Data are mean ± SD. N = 3. * *p* <0.05, ** *p* <0.01, *** *p* <0.001, one-way ANOVA with Bonferroni’s post hoc test. Beta cell mass was calculated as follows: beta cell mass (mg) = (beta cell area ∕ pancreatic area) × pancreas weight (mg). PS, physiological saline; DMSO, dimethyl sulfoxide; STZ streptozotocin; 3PO, 3-(3-pyridinyl)-1-(4-pyridinyl)-2-propen-1-one.


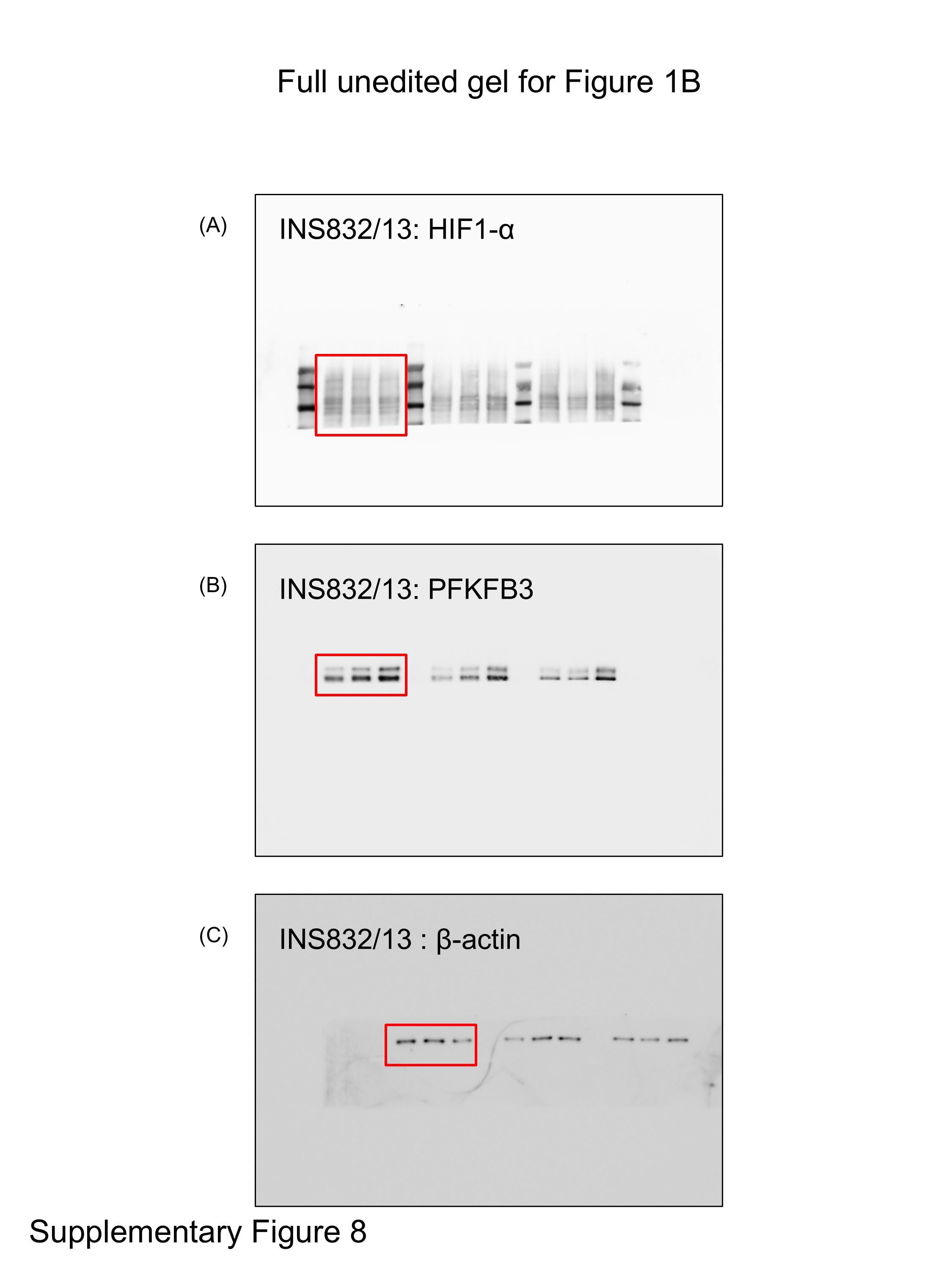


**Supplementary Figure S8. Uncropped western blot images for Figure 1B.**

Boxes indicate the cropped regions in Figure 1B. Each uncropped corresponds to HIF1α (A), PFKFB3 (B), and β-actin (C), respectively. β-actin (C) was used as the internal control for all gels of Supplementary Figure S8. HIF1α, PFKFB3 and β-actin were detected on the same gel; however, different exposure conditions may have been applied to each blot.


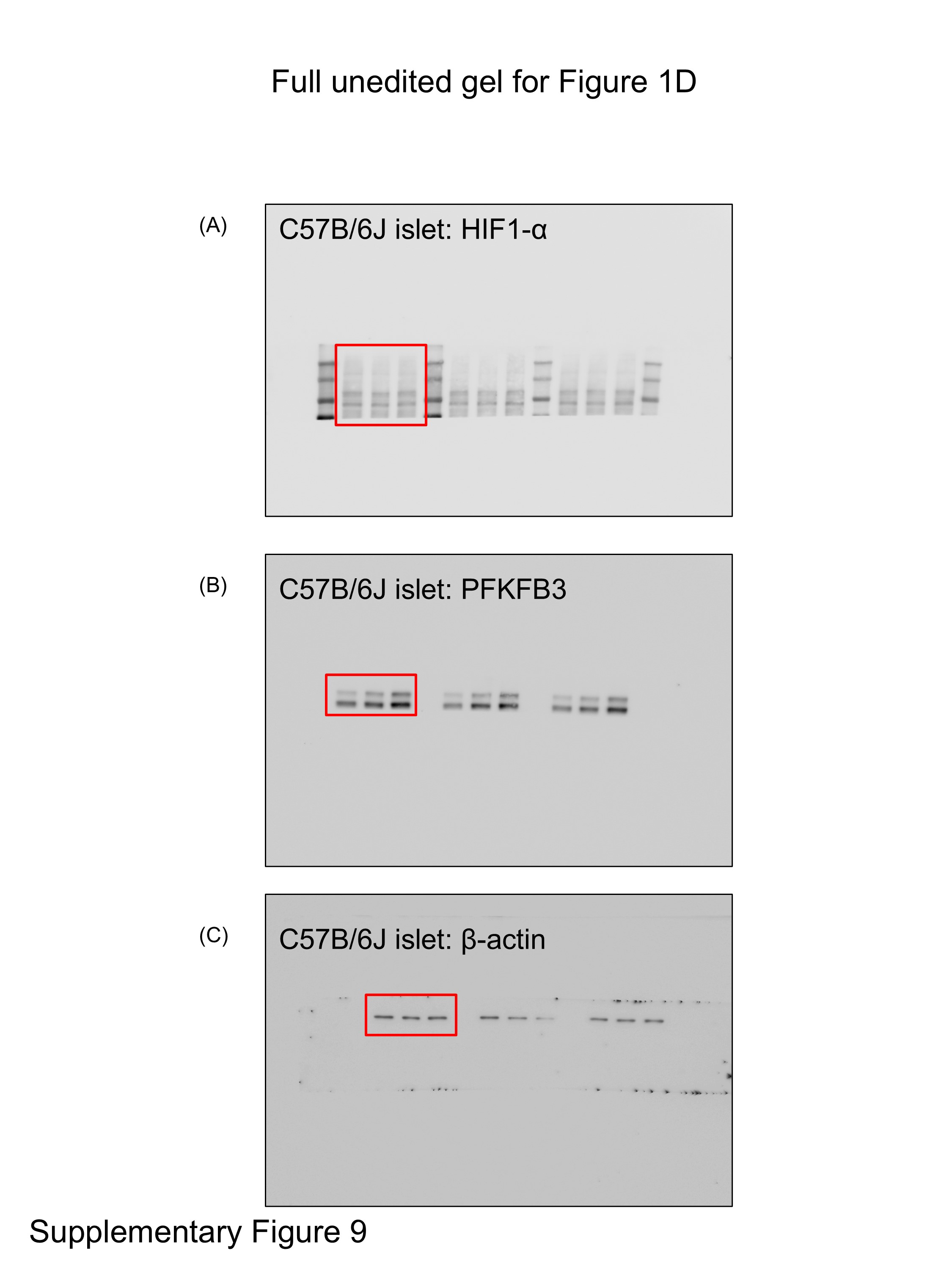


**Supplementary Figure S9. Uncropped western blot images for Figure 1D.**

Boxes indicate the cropped regions in Figure 1D. Each uncropped corresponds to HIF1α (A), PFKFB3 (B), and β-actin (C), respectively. β-actin (C) was used as the internal control for all gels of Supplementary Figure S9. HIF1α, PFKFB3 and β-actin were detected on the same gel; however, different exposure conditions may have been applied to each blot.


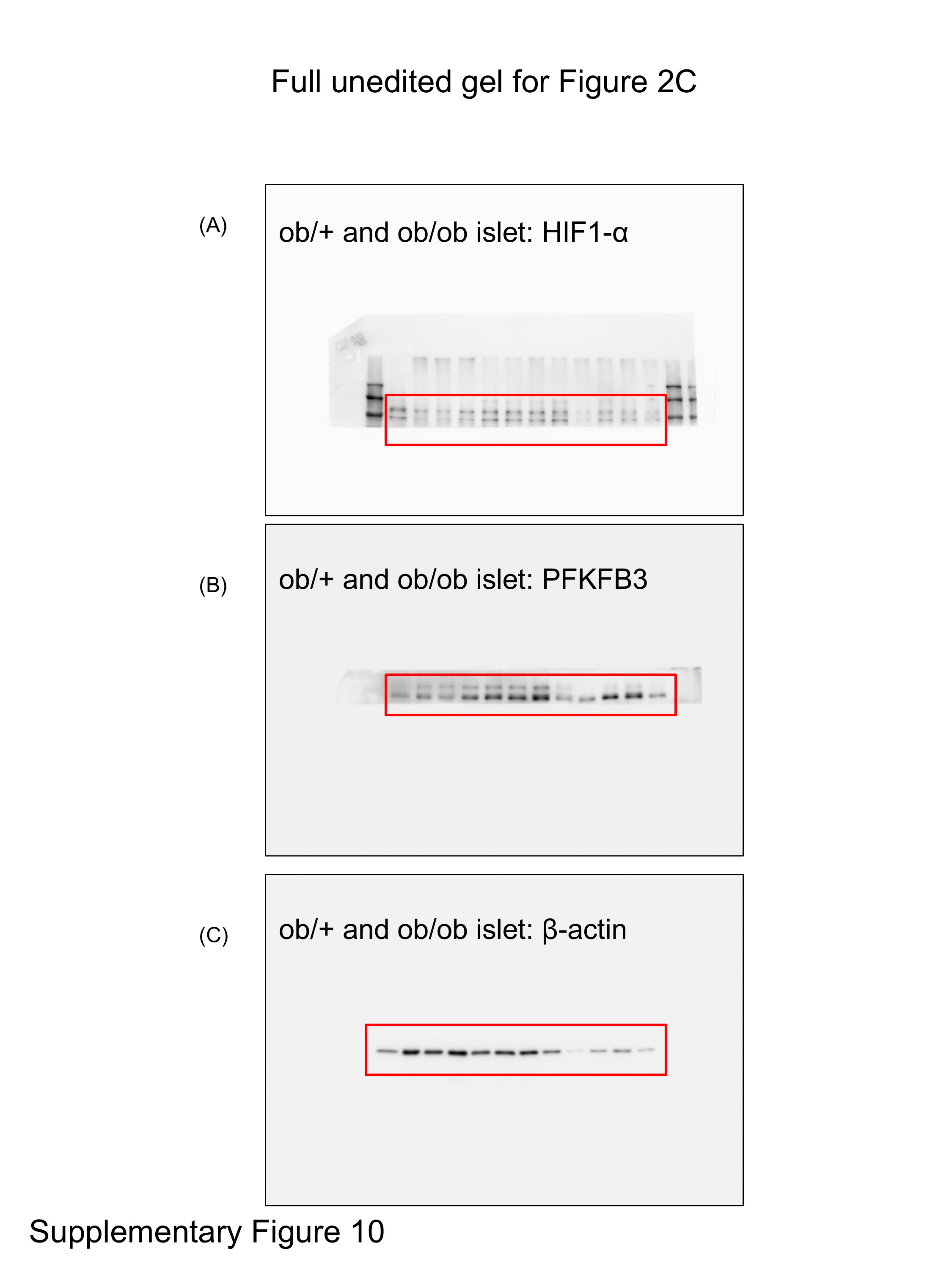


**Supplementary Figure S10. Uncropped western blot images for Figure 2C.**

Boxes indicate the cropped regions in Figure 2C. Each uncropped corresponds to HIF1α (A), PFKFB3 (B), and β-actin (C), respectively. β-actin (C) was used as the internal control for all gels of Supplementary Figure S10. HIF1α, PFKFB3 and β-actin were detected on the same gel; however, different exposure conditions may have been applied to each blot.


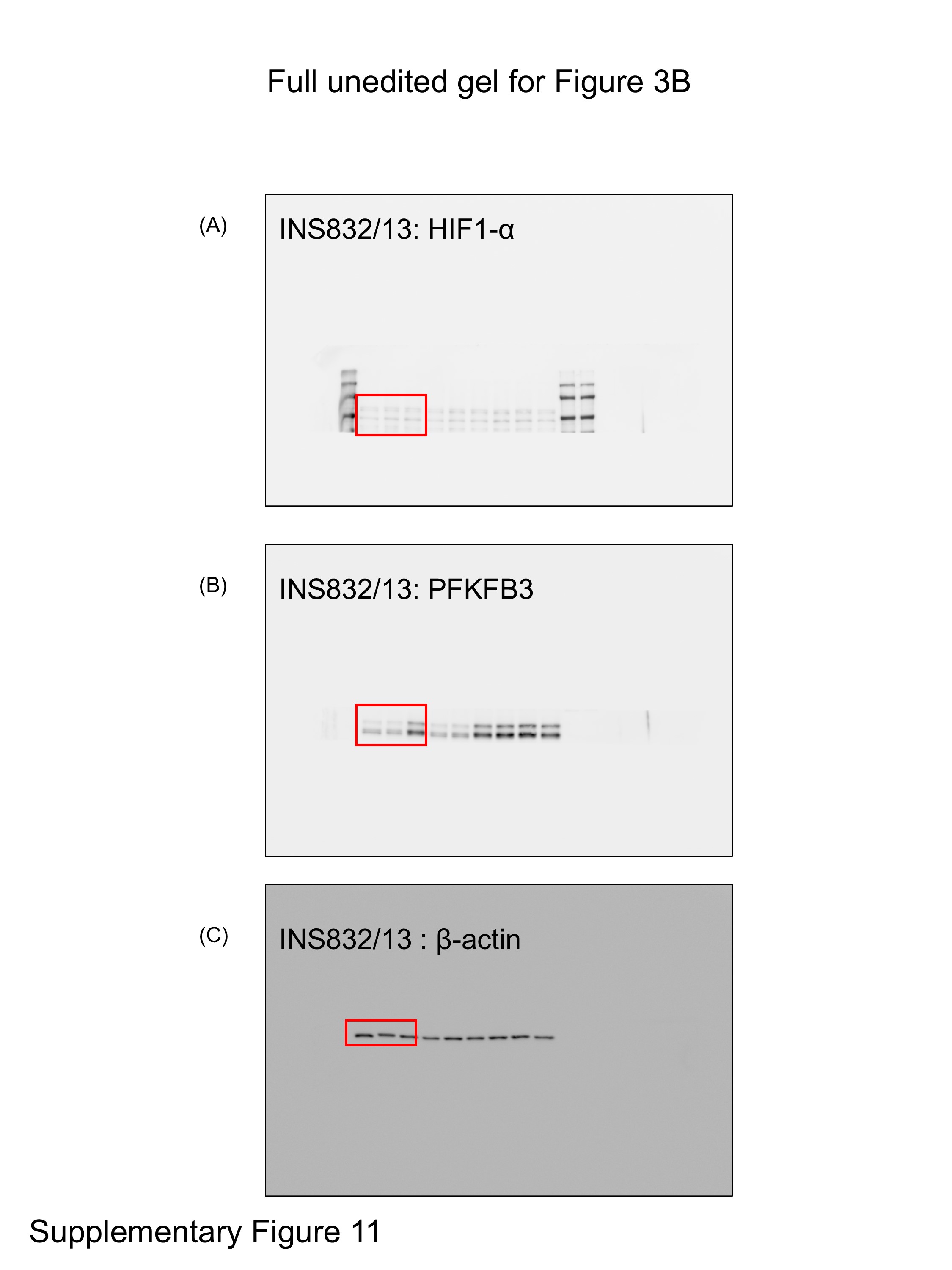


**Supplementary Figure S11. Uncropped western blot images for Figure 3B.**

Boxes indicate the cropped regions in Figure 3B. Each uncropped corresponds to HIF1α (A), PFKFB3 (B), and β-actin (C), respectively. β-actin (C) was used as the internal control for all gels of Supplementary Figure S11. HIF1α, PFKFB3 and β-actin were detected on the same gel; however, different exposure conditions may have been applied to each blot.


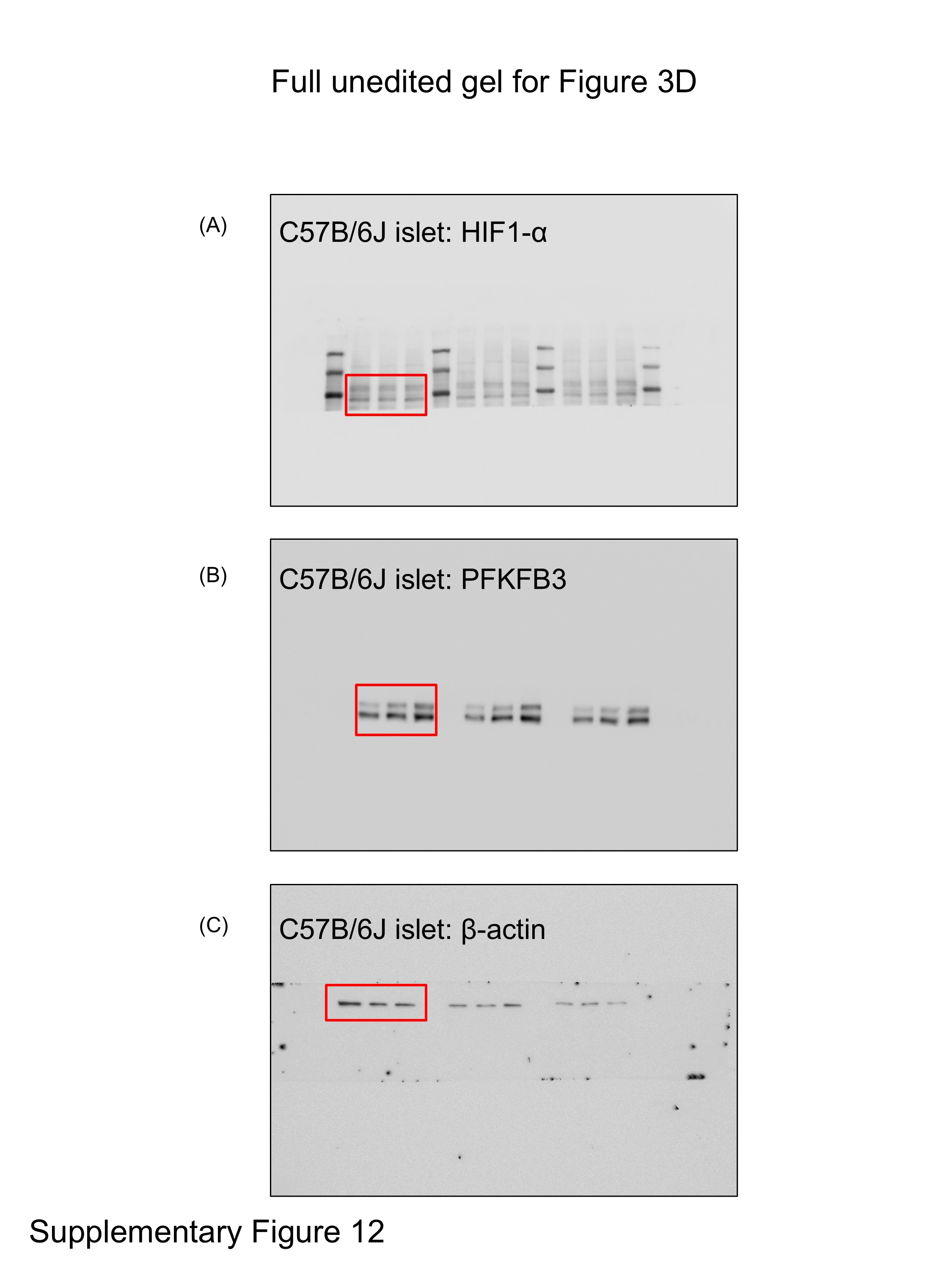
 **Supplementary Figure S12. Uncropped western blot images for Figure 3D.**

Boxes indicate the cropped regions in Figure 3D. Each uncropped corresponds to HIF1α (A), PFKFB3 (B), and β-actin (C), respectively. β-actin (C) was used as the internal control for all gels of Supplementary Figure S12. HIF1α, PFKFB3 and β-actin were detected on the same gel; however, different exposure conditions may have been applied to each blot.


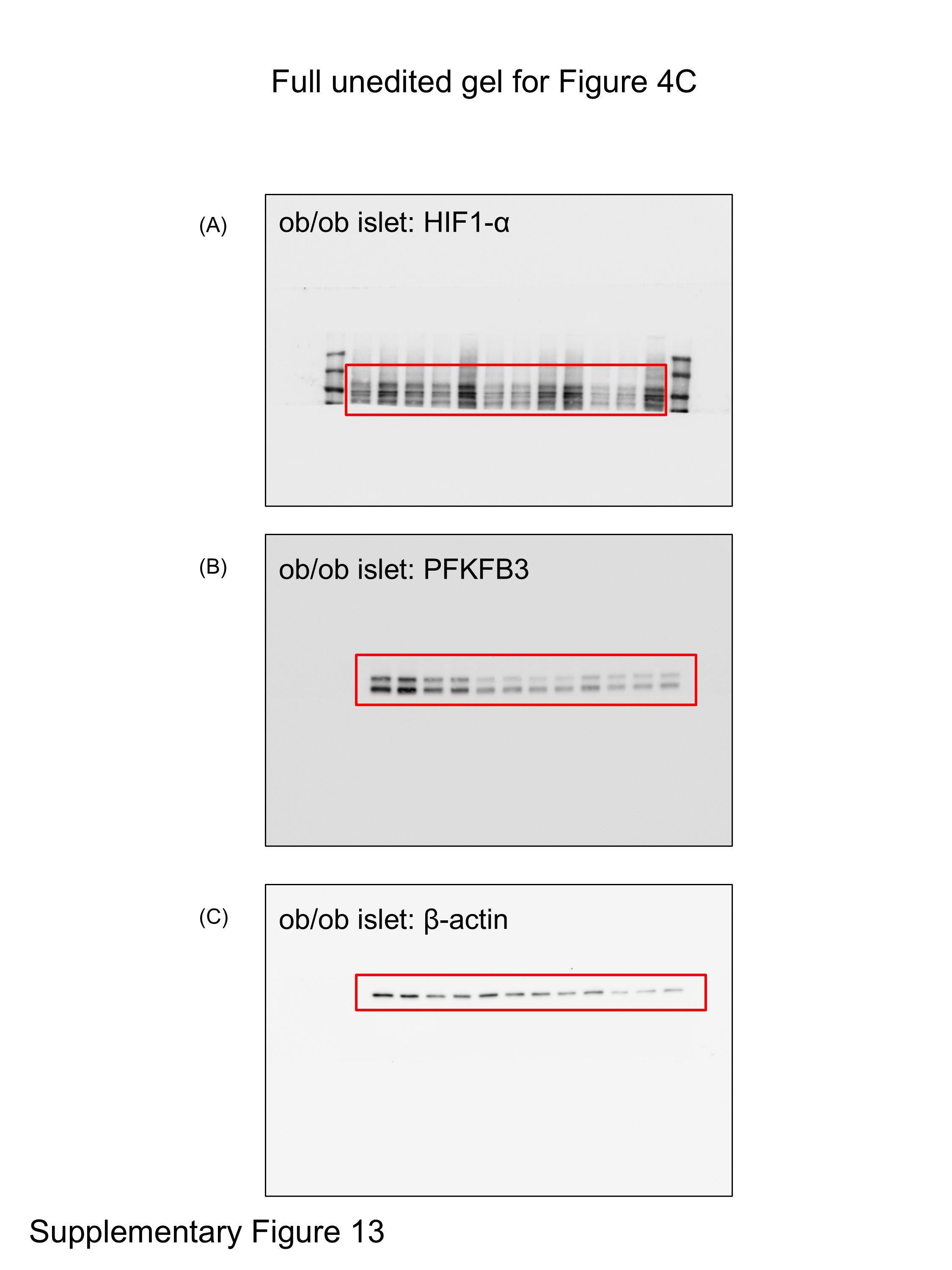
 **Supplementary Figure S13. Uncropped western blot images for Figure 4C.**

Boxes indicate the cropped regions in Figure 4C. Each uncropped corresponds to HIF1α (A), PFKFB3 (B), and β-actin (C), respectively. β-actin (C) was used as the internal control for all gels of Supplementary Figure S13. HIF1α, PFKFB3 and β-actin were detected on the same gel; however, different exposure conditions may have been applied to each blot.


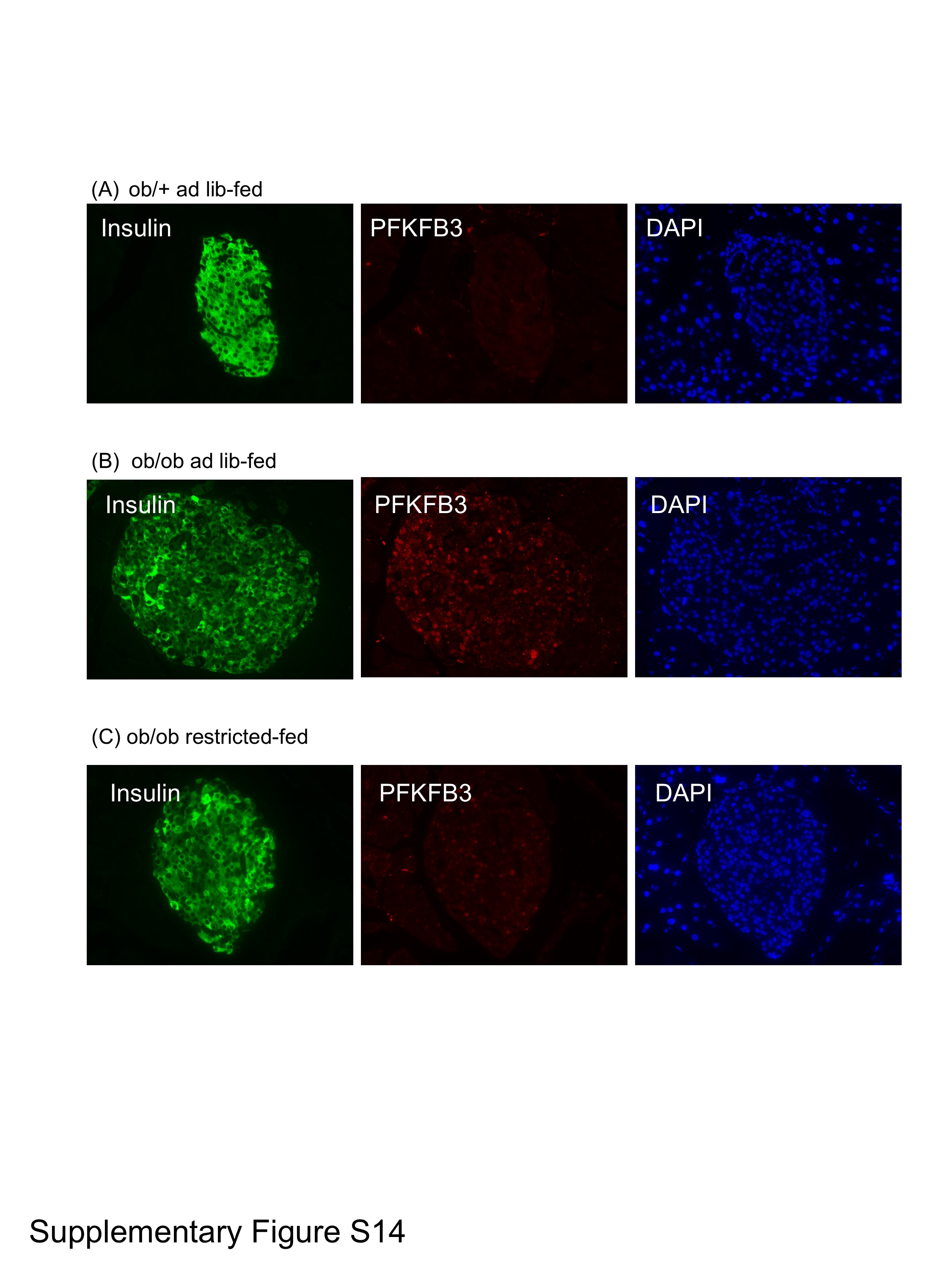


**Supplementary Figure S14. Split-channel immunofluorescence images corresponding to Figure 2E.**
Representative images of pancreatic islets from ob/+ and ob/ob mice under ad libitum or restricted feeding conditions. Individual fluorescence signals are shown for insulin (green), PFKFB3 (red), and nuclei stained with DAPI (blue). Merged images with scale bars are presented in Figure 2E. Scale bars are not shown in these split-channel images. PFKFB3, 6-phosphofructo-2-kinase/fructose-2,6-biphosphatase 3.


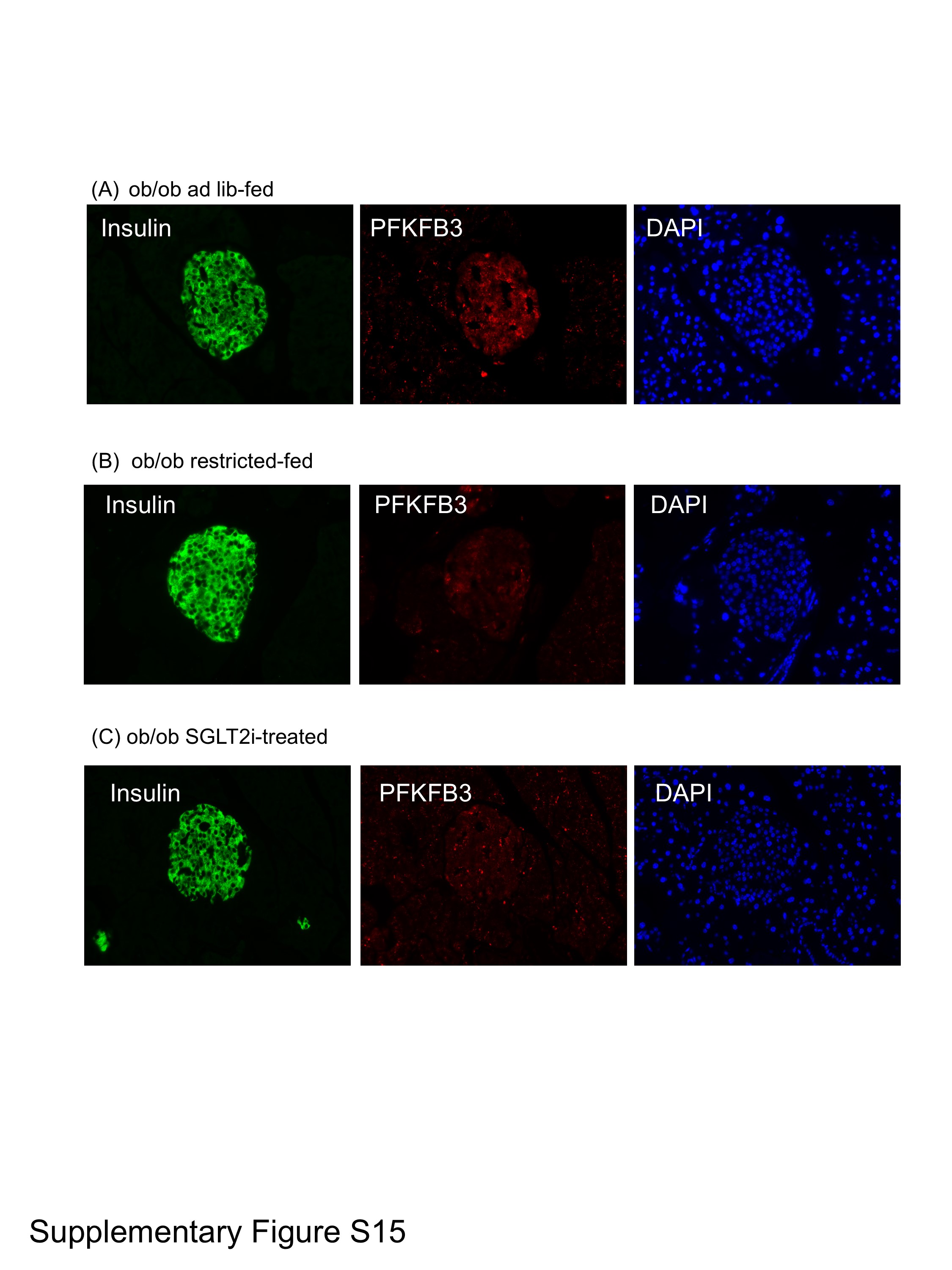


**Supplementary Figure S15. Split-channel immunofluorescence images corresponding to Figure 4E.**
Representative images of pancreatic islets from ob/ob mice under ad libitum feeding, restricted feeding, or treatment with the SGLT2 inhibitor tofogliflozin. Immunofluorescence signals are shown for insulin (green), PFKFB3 (red), and DAPI (blue). Merged images with scale bars are presented in Figure 4E. Scale bars are not shown in these split-channel images. PFKFB3, 6-phosphofructo-2-kinase/fructose-2,6-biphosphatase 3.


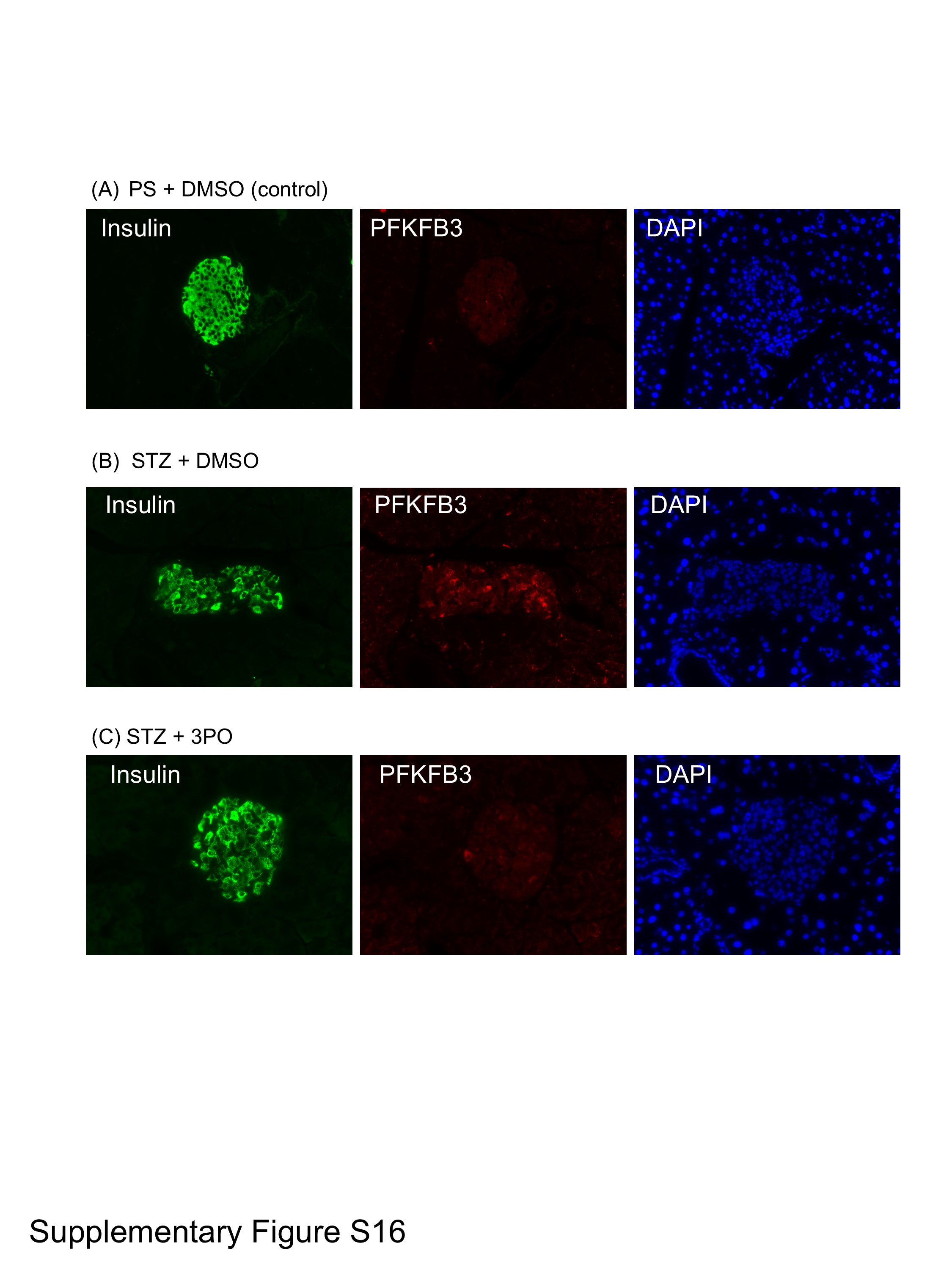


**Supplementary Figure S16. Split-channel immunofluorescence images corresponding to Figure 6A.**
Representative images of pancreatic islets from C57BL6/J mice treated with physiological saline (PS + DMSO), streptozotocin (STZ + DMSO), or STZ plus the PFKFB3 inhibitor 3PO (STZ + 3PO). Fluorescence signals for insulin (green), PFKFB3 (red), and DAPI (blue) are shown separately. Merged images with scale bars are presented in Figure 6A. Scale bars are not shown in these split-channel images. PFKFB3, 6-phosphofructo-2-kinase/fructose-2,6-biphosphatase 3.


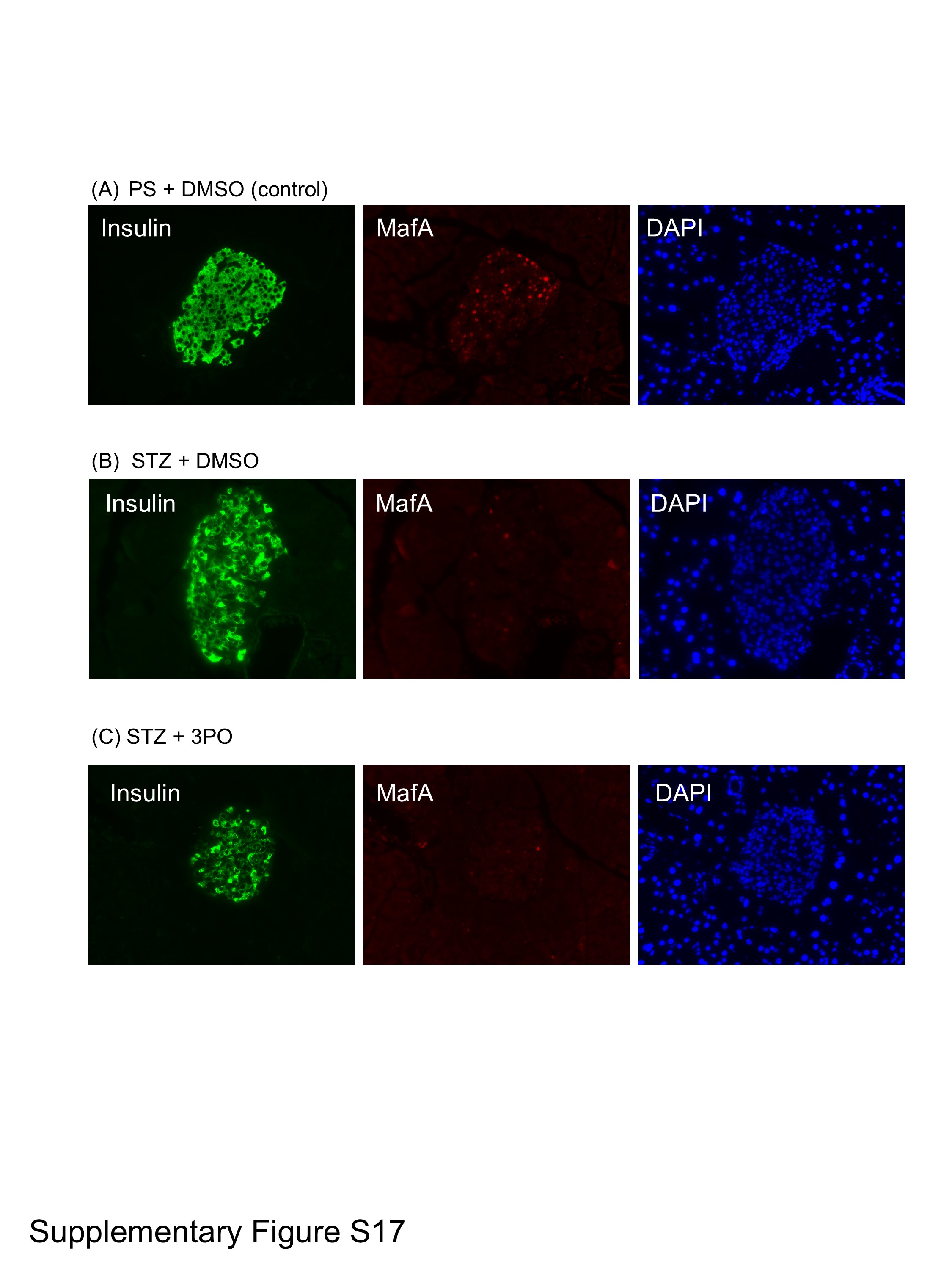


**Supplementary Figure S17. Split-channel immunofluorescence images corresponding to Figure 6E.**
Representative images of pancreatic islets from C57BL6/J mice treated with physiological saline (PS + DMSO), STZ + DMSO, or STZ + 3PO. Individual fluorescence signals are shown for insulin (green), MafA (red), and DAPI (blue). Merged images with scale bars are shown Figure 6E. Scale bars are not shown in these split-channel images. PFKFB3, 6-phosphofructo-2-kinase/fructose-2,6-biphosphatase 3.

**Supplementary Table S**1. Primary and secondary antibodies used in immunofluorescence

| **Primary antibodies** | | | | |
| --- | --- | --- | --- | --- |
| **Antigen** | **Species** | **Dilution** | **Manufacturer** | **Catalog#** |
| Insulin | Guinea pick | 1:1 | Dako | IR002 |
| PFKFB3 | Rabbit | 1:400 | abcam | ab18186 |
| MafA | Rabbit | 1:250 | Fortis Life Sciences | IHC-00352 |
| **Secondary antibodies** | | | | |
| Guinea pig IgG  (Alexa Fluor 488) | Goat | 1:200 | Life Technologies | A11073 |
| Rabbit IgG  (Alexa Fluor 594) | Goat | 1:200 | Life Technologies | A11012 |

**Supplementary Table S2. The primer s**equences used for real-time quantitative PCR

| Gene (Forward/Reverse) | Sequence |
| --- | --- |
| Rat |  |
| β actin forward | CTCTCAGCTGTGGTGGTGAA |
| β actin reverse | GTCGTACCACTGGCATTGTG |
| PFKFB3 forward | CACGGCGAGAATGAGTACAA |
| PFKFB3 reverse | TTCAGCTGACTGGTCCACAC |
| MafA forward | CTTCAGCAAGGAGGAGGTCATC |
| MafA reverse | GCGTAGCCGCGGTTCTT |
| Pdx-1 forward | AGGAGGTGCATACGCAGCAG |
| Pdx-1 reverse | GAGGCCGGGAGATGTATTTGTT |
| Mouse |  |
| β actin forward | ATATCGCTGCGCTGGTCTGTC |
| β actin reverse | AGCACAGCCTGGATGGCTAC |
| PFKFB3 forward | AGAACTTCCACTCTCCCACCC |
| PFKFB3 reverse | AGGGTAGTGCCCATTGTTGAA |
| MafA forward | CTTCAGCAAGGAGGAGGTCATC |
| MafA reverse | GCGTAGCCGCGGTTCTT |
| Pdx-1 forward | CTCCGGACATCTCCCCATAC |
| Pdx-1 reverse | ACGGGTCCTCTTGTTTTCCT |

**Supplementary Table S3. Primary and secondary antibodies used in western blot**

| **Primary antibodies** | | | | |
| --- | --- | --- | --- | --- |
| **Antigen** | **Species** | **Dilution** | **Manufacturer** | **Catalog#** |
| HIF1-alpha | Rabbit | 1:1000 | abcam | ab2185 |
| PFKFB3 | Rabbit | 1:1000 | abcam | ab181861 |
| LDHA | Rabbit | 1:1000 | Cell signaling | 2012S |
| HK1 | Rabbit | 1:1000 | Cell signaling | 2024T |
| GAPDH | Rabbit | 1:1000 | Cell signaling | 2118S |
| Beta-actin | Goat | 1:1000 | Santa Cruz Biotechnology | sc-1615 |
| **Secondary antibodies** | | | | |
| Goat anti Rabbit HRP | Goat | 1:10000 | Bio-Rad Laboratories | 170-6515 |
| Donkey anti Goat HRP | Donkey | 1:10000 | Santa Cruz Biotechnology | sc-2020 |
